# Digital Bioimpedance for Physical Activity Detection in Type-2 Diabetes: Quasi-Experimental Validation Study

**DOI:** 10.2196/83768

**Published:** 2025-12-16

**Authors:** Akira Kimura, Shinobu Onozawa, Takayuki Ogiwara, Marwan El Ghoch

**Affiliations:** 1 Graduate School of Health Science, Gunma Paz University, 1-7-1 Tonyamachi, Takasaki, 370-0006, Japan, 81 273653366, 81 273880865; 2 Division of Endocrinology and Diabetes, Internal Medicine, Saiseikai Maebashi Hospital, Maebashi, Japan; 3 Department of Biomedical, Metabolic and Neural Sciences, University of Modena and Reggio Emilia, Modena, Italy

**Keywords:** digital health, bioelectrical impedance, diabetes mellitus, type 2, exercise therapy, monitoring, physiologic, primary health care, built environment, hemoglobin A, glycosylated

## Abstract

**Background:**

Primary care diabetes management lacks objective, scalable methods for continuous physical activity surveillance. Bioelectrical impedance analysis (BIA), routinely collected in diabetes care, offers untapped potential as an automated digital biomarker but requires validation for behavioral phenotyping.

**Objective:**

This study aims to evaluate the feasibility and predictive validity of multifrequency bioimpedance for physical activity detection and its association with glycemic control in type 2 diabetes.

**Methods:**

This was a pragmatic quasi-experimental study using temporal allocation across three 4-month periods (January 2021-July 2023) in a Japanese primary care clinic, including comprehensive tracking with BIA-guided counseling (n=65), partial tracking (n=31), and standard care (n=100). Adults with type 2 diabetes (hemoglobin A_1c_ [HbA_1c_] 7.0%‐10.0%) underwent monthly segmental multifrequency BIA. The primary outcome was HbA_1c_ <7% at 4 months. Intervention-outcome associations were examined using chi-square trend tests and multivariable logistic regression adjusted for baseline HbA_1c_, the Walk Score (0‐100), and medication indicators. To assess temporal confounding, we conducted ANCOVA on 4-month HbA_1c_ with baseline adjustment (age and BMI added in sensitivity analyses). Effect modification by built environment was tested via Walk Score×Intervention interaction. Predictive validity of left-arm 50-kHz reactance was assessed using area under receiver operating characteristic curve with 95% CI via 10-fold cross-validation.

**Results:**

Among 196 participants, the baseline characteristics (age, BMI, HbA_1c_, diabetes duration, and medications) did not differ across periods (all *P*>.05). HbA_1c_ <7% achievement showed a gradient: 80% (52/65) comprehensive, 58% (18/31) partial, and 56% (56/100) standard care (*χ*²_4_ for trend=14.23; *P*<.001). ANCOVA of 4-month HbA1c (baseline-adjusted) showed no linear period trend (*P*=.25). A significant Walk Score×Intervention interaction was observed (β per 10-point Walk Score=−.55; 95% CI −1.03 to −0.06; *P*=.028), indicating differential effectiveness by neighborhood walkability. Left-arm 50-kHz reactance predicted target achievement (adjusted odds ratio per 1-SD increase =3.04; 95% CI 1.86‐4.97; *P*<.001; area under receiver operating characteristic curve=0.847, 95% CI 0.784‐0.910). Among achievers, reactance change correlated with HbA1c change (r=−0.392; *P*=.032) but not among nonachievers (r=−0.089; *P*=.54). After the inverse probability weighting was stabilized, each 1-SD increase in left-arm reactance was associated with a 12.1 percentage-point higher probability of target achievement (95% CI 5.2%‐19.0%).

**Conclusions:**

This pragmatic implementation study demonstrates that automated BIA is feasible for routine diabetes care and suggests potential as a digital biomarker of activity-related glycemic control. While temporal allocation precludes definitive causal inference, and findings should be interpreted as associational, the observed Walk Score moderation and bioimpedance-HbA_1c_ dose-response patterns are consistent with behavioral mechanisms rather than pure confounding. Left-arm reactance warrants randomized validation as a scalable, passive surveillance tool for precision diabetes management.

## Introduction

### The Digital Health Revolution in Exercise Tracking

The advent of digital health technologies has transformed chronic disease management, with diabetes care positioned at the forefront of this revolution [1,2]. Mobile health apps, wearable devices, and digital biomarkers now offer unprecedented opportunities for real-time patient monitoring and personalized intervention strategies [3-7]. However, despite significant advances in glucose monitoring technologies, exercise compliance assessment—a cornerstone of diabetes management—remains predominantly dependent on subjective self-reporting methods that lack the precision and objectivity demanded by contemporary precision medicine approaches [8,9]. Current digital health ecosystems for diabetes management excel in glucose tracking but demonstrate a critical deficiency in objective exercise assessment [1,10,11]. While continuous glucose monitors provide minute-by-minute glycemic data, exercise compliance monitoring continues to rely on patient diaries, smartphone step counters, or intermittent subjective assessments during clinical encounters. This disparity creates a fundamental imbalance in the digital health toolkit available to primary care providers, who must make evidence-based treatment decisions regarding exercise therapy optimization without access to reliable, objective compliance data [12].

### Critical Gaps in Primary Care Exercise Tracking

Type 2 diabetes affects over 460 million individuals worldwide, with substantial morbidity, mortality, and health care costs [13-16]. Despite therapeutic advances, only 50% to 60% of patients achieve hemoglobin A_1c_ (HbA_1c_) targets in real-world practice, creating urgent challenges for primary care diabetes management [17,18].

In primary care settings, exercise therapy and dietary modification are universally recommended as first-line interventions [19-24]; however, the digital infrastructure supporting these recommendations remains incomplete. While dietary tracking applications and digital nutrition platforms have achieved widespread adoption, exercise monitoring capabilities lag significantly behind, creating an asymmetric digital health ecosystem that inadequately supports comprehensive lifestyle intervention strategies [3,9].

Traditional methods of monitoring exercise compliance, including patient self-reports, activity diaries, and subjective assessments, are notoriously unreliable and provide insufficient objective data for clinical decision-making [12]. This monitoring deficit creates a critical gap in primary care: physicians cannot provide appropriately tailored exercise therapy guidance without reliable evidence of patient exercise implementation [25].

### The Challenge of Exercise Tracking Through Body Composition Analysis

Self-care management could potentially be enhanced through objective body composition assessment as evidence of exercise implementation. Bioelectrical impedance analysis (BIA) offers promise for detecting exercise-induced physiological changes, providing objective markers that could inform clinical decision-making [26,27]. However, fundamental uncertainties regarding optimal measurement parameters severely limit clinical use [28].

Body composition assessment reveals diverse reactance frequency characteristics across the spectrum from 5 kHz to 250 kHz, with each frequency potentially providing different physiological information [26,29]. Similarly, anatomical measurement sites—from individual limbs to whole-body assessment—may exhibit differential sensitivity to exercise-induced changes [30,31]. The critical clinical question remains: which specific frequency and anatomical site combination most reliably reflects exercise implementation and predicts treatment success in real-time tracking applications?

### The Problem of Contradictory Literature

Recent systematic evaluations reveal the extent of contradictions in bioelectrical impedance research [28,32]. Studies have shown conflicting results regarding which body segments provide optimal metabolic information, with some studies favoring upper extremity measurements and others showing no regional differences [30,33]. The comprehensive literature examining body composition methodologies demonstrates the need for standardized approaches [34].

We propose that these contradictions reflect a previously unrecognized phenomenon: culturally imposed localized inactivity. Across global populations, food preparation and consumption practices create systematic asymmetric limb use patterns that represent “manufactured” localized disuse. For instance, cultures using utensils consistently favor one hand for manipulation while the other provides stabilization, creating daily repetitive asymmetric loading patterns. This cultural specificity of body usage represents a unique form of environmental laterality conditioning that has been overlooked in previous bioimpedance research.

### Unified Theoretical Framework for Population-Specific Tracking

We hypothesize that anatomical specificity of metabolic biomarkers depends on the degree of baseline body usage asymmetry in the studied population. This unified framework predicts the following:

- High-asymmetry populations (consistent preferential use of one limb): The less-used limb demonstrates preferential metabolic responsiveness due to greater adaptive capacity for exercise tracking.
- Moderate-asymmetry populations (mild preferential use): The dominant limb may show preferential responsiveness due to higher baseline metabolic activity.
- Low-asymmetry populations (balanced bilateral use): Bilateral symmetry or whole-body measures provide optimal sensitivity.
- Mixed populations (heterogeneous usage patterns): No consistent anatomical patterns emerge, diluting site-specific tracking effects.

This framework enables systematic reconciliation of contradictory findings while providing practical guidance for optimizing digital biomarker protocols in diabetes care.

### Study Objectives

Given this theoretical framework and the documented need for objective physical activity monitoring in primary care diabetes management, this study aimed to: (1) validate a digital bioimpedance protocol for objective physical activity detection in Japanese adults with type 2 diabetes, (2) identify population-specific anatomical sites demonstrating optimal sensitivity for activity tracking, and (3) evaluate the clinical use of this framework for predicting glycemic target achievement in real-world primary care settings. We hypothesized that left-arm 50-kHz reactance would demonstrate superior predictive value compared to other anatomical sites, consistent with high-asymmetry population patterns.

## Methods

### Study Design and Participants

This quasi-experimental study follows the STROBE (Strengthening the Reporting of Observational Studies in Epidemiology) guidelines (Checklist 1) for reporting observational studies. The study used temporal allocation (sequential enrollment by time period) rather than individual randomization, which is acknowledged as a significant limitation affecting causal inference. For more details, see the “Study Limitations” section.

This quasi-experimental, temporally allocated observational study was conducted at Saiseikai Maebashi Hospital, Japan, from January 2022 to July 2023. The study was approved by the institutional review board of Gunma Paz University (IRB No PAZ23-9; approval date: June 15, 2021) and was retrospectively registered in the UMIN Clinical Trials Registry (UMIN000058452; registration date: July 14, 2025). The rationale for retrospective registration is disclosed transparently given the observational design. This study followed the STROBE guidelines for reporting observational studies.

Adults aged 20 to 80 years with type 2 diabetes (HbA_1c_ 7.0%‐10.0%) were recruited through the endocrinology clinic. The study used an opt-out consent process approved by the institutional review board; patients could decline participation at any time. Japanese adults were selected as an ideal population for testing the unified framework, given consistent asymmetric upper-limb use in daily activities.

### Eligibility Criteria

#### Inclusion Criteria

The inclusion criteria are as follows: adults aged 20‐80 years with type 2 diabetes, baseline HbA_1c_ (7.0%‐10.0%), available segmental BIA at baseline, and follow-up HbA_1c_ at 2 and 4 months.

#### Exclusion Criteria

The exclusion criteria are as follows: type 1 or secondary diabetes, pregnancy or lactation, serious liver disease (alanine aminotransferase >3×upper limit of normal), heart failure (New York Heart Association class III–IV); dialysis or estimated glomerular filtration rate <30 mL min^–1^ 1.73 m^–^², implanted pacemaker or implantable cardioverter-defibrillator or other contraindication to BIA, generalized edema requiring active diuretic titration, and patients who declined participation via the opt-out notice.

#### Participant Characteristics and Medication Profile

At baseline, participants had a mean age of 58.7 (SD 11.2) years, mean BMI of 26.4 (SD 4.1) kg m^–^², and mean diabetes duration of 8.3 (SD 5.6) years. Diabetes medications included metformin (n=176, 91%), sulfonylureas (n=87, 45%), dipeptidyl peptidase-4 inhibitors (n=112, 58%), sodium-glucose cotransporter 2 inhibitors (n=64, 33%), and insulin (n=31, 16%). Diuretic use was documented in 28 (14%) participants. Participants were instructed to maintain stable medication regimens throughout the study period unless medically necessary adjustments were required; such changes were documented and accounted for in sensitivity analyses. No participants had clinical evidence of peripheral edema, acute illness, or recent hospitalization at baseline assessment. Standard hydration instructions (4-h fast and avoid strenuous exercise 24 h prior) were provided for all bioimpedance measurements to minimize measurement variability from acute hydration status.

### Digital Exercise Tracking Protocol: Exercise Prescription Specifics

The comprehensive intervention (period A) included individualized exercise prescriptions based on baseline fitness assessment. Recommended activities included brisk walking (primary modality), cycling, swimming, or home-based calisthenics, prescribed at moderate intensity (50%‐70% of age-predicted maximum heart rate or 12‐14 on the Borg Rating of Perceived Exertion scale). Target duration was 30 minutes per session, 5 days per week, with gradual progression from baseline activity levels. Participants received written exercise logs and were instructed to record activity type, duration, and perceived exertion. The partial intervention (period B) received standard verbal advice about exercise benefits without individualized prescriptions, activity logs, or follow-up counseling. Standard care (period C) received routine diabetes education consistent with usual clinic practice, which included general encouragement for physical activity but no structured exercise prescription or monitoring.

### Digital Bioimpedance Assessment Protocol

#### Overview

All bioelectrical impedance measurements were obtained with a multifrequency, Food and Drug Administration (FDA) 510(k)-cleared analyzer (InBody 770; InBody Co; K123228 and K141483) that provides standardized, operator-independent measurements suitable for clinical implementation [31]. The device uses eight-point tactile electrodes and automatic self-calibration to ensure consistency across operators and time points—critical for sustainable primary care implementation [26,29].

The InBody 770 uses an 8-point tactile electrode system with separate current-injecting and voltage-sensing electrodes for each limb segment, implementing a tetrapolar measurement approach that minimizes contact impedance artifacts. This configuration uses thumb and palm electrodes for each hand, and heel and forefoot electrodes for each foot, with measurements obtained in a standardized standing position. The device applies alternating current at multiple frequencies (1 kHz, 5 kHz, 50 kHz, 250 kHz, 500 kHz, and 1000 kHz) through the current electrodes while simultaneously measuring voltage drops across the sensing electrodes, calculating impedance from the Ohm law (Z=V/I). Segmental measurements are obtained sequentially for each body segment (right arm, left arm, trunk, right leg, and left leg) within a total measurement duration of approximately 60 seconds per assessment.

#### Motion Artifact Mitigation

The primary concern regarding bioimpedance measurement validity is motion artifact, particularly during or immediately after physical activity. To address this critical limitation, our protocol implemented several complementary strategies. First, all bioimpedance measurements were obtained during standardized static conditions—participants stood motionless in the designated measurement position for 30 seconds prior to measurement initiation, allowing hemodynamic stabilization and eliminating movement-related noise. The standing position with arms abducted at approximately 15 degrees from the body trunk minimizes interlimb contact while maintaining postural stability.

Second, the InBody 770’s tetrapolar electrode configuration inherently reduces motion artifact through its separation of current-injecting and voltage-sensing electrodes; this design ensures that contact impedance variations (a primary source of motion artifact) affect only the high-impedance voltage-sensing pathway, which contributes negligibly to total measured impedance.

Third, the device’s internal quality control algorithms automatically detect and flag measurements with excessive impedance variance (coefficient of variation >5% across repeated cycles within a single measurement session), prompting remeasurement when unstable contact is detected.

Fourth, our measurement protocol deliberately avoided assessments during or immediately after physical activity. Bioimpedance measurements were obtained at 3 standardized time points (baseline, 2 mo, and 4 mo) during routine clinical visits, with participants instructed to sit quietly for at least 10 minutes prior to assessment. This approach fundamentally differs from real-time exercise monitoring; rather, we leveraged the physiological principle that habitual physical activity induces detectable and persistent changes in tissue bioimpedance properties (reflecting fluid redistribution, muscle adaptation, and metabolic remodeling) that remain measurable during subsequent static assessments, analogous to how glycated hemoglobin reflects long-term glycemic exposure rather than instantaneous glucose levels. The distinction between measuring bioimpedance during exercise (susceptible to severe motion artifact) versus measuring bioimpedance changes attributable to habitual exercise patterns (measured under static conditions) is fundamental to our study design and has been successfully employed in previous investigations of training-induced adaptations.

#### Data Quality Control and Processing

Raw bioimpedance data underwent systematic quality assessment before analysis. First, device-level quality control flagged measurements with impedance values outside physiologically plausible ranges (resistance <200 Ω or >800 Ω for limb segments; reactance <20 Ω or >100 Ω) for review and potential remeasurement. Second, longitudinal consistency checks identified implausible changes between timepoints (>20% change in resistance or reactance within a 2-mo period in the absence of major clinical events), triggering verification of measurement conditions and participant status. Third, we excluded measurements obtained during documented acute illness, recent surgery, or major fluid status alterations (such as hospitalization for heart failure or dialysis initiation), as these conditions induce bioimpedance changes unrelated to habitual physical activity patterns.

The InBody 770 automatically calculates both resistance (R) and reactance (Xc) components from the measured complex impedance at each frequency. Resistance reflects the opposition to current flow through intra- and extracellular fluids, while reactance reflects the capacitive properties of cell membranes and tissue interfaces. For this analysis, we focused on 50-kHz reactance as the primary biomarker, as this frequency provides optimal sensitivity to cellular membrane properties and intracellular fluid distribution, which are hypothesized to be preferentially altered by physical activity in skeletal muscle tissue. All exported data values represent the mean of 3 consecutive measurement cycles automatically performed by the device during each assessment session, with the device internally validating cycle-to-cycle consistency before data export.

To ensure measurement reproducibility, all assessments were conducted under standardized conditions: participants were instructed to fast for at least 4 hours, avoid strenuous exercise for 24 hours prior, maintain usual medication including diuretics, and measurements were performed between 9 AM and 11 AM. Daily device calibration was performed according to manufacturer specifications. Test-retest reliability in a pilot sample (n=20) showed a coefficient of variation <2.5% for 50 kHz reactance measurements.

Digital data were automatically captured and stored using the device’s integrated data management system, eliminating transcription errors and enabling real-time data analysis [1,3]. Measurements included impedance values at 1 kHz, 5 kHz, 50 kHz, 250 kHz, and 500 kHz frequencies across 5 body segments (right arm, left arm, trunk, right leg, and left leg), generating a comprehensive digital bioimpedance profile for each participant [26,30].

### Assessment of Hand Dominance and Use Patterns

Hand dominance and use asymmetry were assessed using the Edinburgh Handedness Inventory administered at baseline. Participants reported: (1) writing hand preference, (2) hand used for complex manipulations during eating with utensils (chopsticks, spoon, and fork), and (3) age when these patterns became established. Data were collected via standardized questionnaire.

### Real-Time Data Integration Framework

#### Overview

Environmental walkability data were digitally assessed using the Walk Score algorithm, which provides standardized, reproducible neighborhood assessments based on proximity to amenities and infrastructure [35-38]. Walk Score has been validated in Japanese settings [39,40]. Scores were obtained using participants’ residential addresses (verified at enrollment), calculated within 800-meter radius using the Walk Score application programming interface (API; accessed between January 2022 and February 2022) under standard academic use terms. This digital approach eliminates subjective environmental assessment variability while providing immediately available data that can be integrated into clinical decision-making workflows [41,42].

#### Data Processing Timeline

The term “real-time” in our framework refers to the immediate availability of bioimpedance data at the point of care, enabling clinical decision-making during the same visit. The InBody 770 device completes measurement and generates output reports within approximately 60 seconds of measurement initiation. These data were immediately available to clinicians via the device display and integrated electronic medical record system, with no additional processing delay. This contrasts with traditional body composition assessment methods requiring off-site laboratory analysis or specialized technician interpretation. The data integration architecture enabled clinicians to view bioimpedance trends (current vs baseline values) instantly, facilitating informed discussions about activity patterns and treatment adjustments during the clinical encounter rather than requiring follow-up visits for results review.

### Temporal Allocation Methodology

As registered in UMIN000058452, participants were allocated to treatment groups based on distinct enrollment periods, representing a quasi-experimental design with temporal allocation. This nonrandomized design was chosen for pragmatic implementation feasibility in routine clinical practice as follows:

1. Period A—comprehensive tracking intervention (n=65): participants enrolled between January 2021 and June 15, 2021, received intensive digital tracking–based lifestyle intervention consisting of monthly individual nutritional counseling, bioelectrical impedance monitoring every 2 months with detailed feedback, personalized exercise recommendations based on individual tracking data, and structured diabetes education [43,44].
2. Period B—partial tracking intervention (n=30): participants enrolled between April 2022 and June 2022 received moderate intervention with quarterly body composition monitoring and basic feedback [45].
3. Period C—standard care control group (n=98): participants enrolled between July 2022 and January 2023 received standard diabetes management with routine visits every 3 to 4 months [25,43].

### Outcomes and Measurements

#### Primary Hypothesis and Outcomes

On the basis of the theoretical framework, we hypothesized that left-arm 50 kHz reactance would demonstrate greater predictive value for glycemic outcome achievement in this high-asymmetry population compared to other anatomical sites and frequencies.

The primary outcome is the achievement of good glycemic control (HbA_1c_ <7.0%) at 4 months, assessed as binary outcome [22,23,43].

#### Secondary Outcomes

The secondary outcomes include (1) change in HbA_1c_ from baseline to 4 months (continuous), (2) achievement of clinically meaningful HbA_1c_ reduction (≥0.5%), (3) body composition changes (50 kHz reactance, muscle mass, and body fat percentage), and (4) muscle strength index changes [30,31].

### Statistical Analysis

#### Overview

Sample size calculation was based on detecting a clinically meaningful difference in glycemic control achievement between allocation groups. With an expected 40% success rate in standard care and 65% in the comprehensive intervention group, the target sample size of 193 participants provided 80% power with *α*=.05 [17].

#### Handling of Multiple Comparisons

Given the exploratory nature of testing multiple anatomical sites (5 segments)×frequencies (5 frequencies)=25 comparisons, we applied Benjamini-Hochberg false discovery rate (FDR) correction to control for multiple testing. The pre-specified primary hypothesis (left arm 50 kHz) was tested with adjusted significance threshold.

#### Primary Analysis

Bioelectrical parameters were evaluated for association with primary outcome using multivariable logistic regression, adjusting for prespecified covariates: age, sex, diabetes duration, baseline HbA_1c_, BMI, insulin use, oral antidiabetic medication number, baseline physical activity level, comorbidity count, and Walk Score. Multicollinearity was assessed using variance inflation factors (<3 for all variables).

#### Missing Data

All analyses followed modified intention-to-treat principles, including all 193 enrolled participants. Missing data (5.1% for follow-up HbA_1c_, 3.6% for bioimpedance measures) was handled using multiple imputation by chained equations with 20 imputations. Imputation models included all covariates, baseline values, and auxiliary variables. Convergence was confirmed by trace plots and the Gelman-Rubin statistics (all <1.1).

#### Sensitivity Analyses

To address potential temporal confounding, we conducted: (1) analyses adjusting for enrollment month as categorical variable, (2) analyses stratified by season (winter or spring vs summer), and (3) interrupted time series analysis treating each period as a distinct time segment.

#### Model Validation

Internal validation was performed using 10-fold cross-validation repeated 100 times. Model performance was evaluated using area under receiver operating characteristic curve (AUC), calibration plots, Brier scores, sensitivity, specificity, positive predictive value, and negative predictive value at optimal threshold (determined by the Youden index).

#### Interaction Model

logit{P(goal attainment)} = β₀+β₁·(Left-arm Xc₅₀ per SD)+β₂·(Walk Score per 10 points)+β₃·(Left-arm Xc₅₀×Walk Score)+prespecified covariates (age, sex, diabetes duration, baseline HbA1c, BMI, medication classes, baseline activity, comorbidities, smoking, calendar month). Multiplicity across segment×frequency comparisons was controlled using the Benjamini-Hochberg FDR. The Walk Score×Intervention interaction showed a significant negative coefficient (−0.055, 95% CI −0.103 to −0.006*; P*=.028; see Multimedia Appendix 1 for full model details), indicating enhanced intervention effects in more walkable environments.

#### Statistical Software and Reporting Standards

All statistical analyses were conducted using R (version 4.2.0; R Foundation for Statistical Computing). Two-sided tests were used. Multiplicity was controlled with the Benjamini-Hochberg FDR; adjusted *P*<.05 was considered statistically significant. For brevity, we report adjusted *P* values and key effect sizes with 95% CIs in the main text; full coefficient tables are available upon request. In the abstract, we report only the key estimates.

### Predictive Model Development

The primary predictive analysis employed multivariable logistic regression with HbA_1c_ <7% achievement as the outcome. Candidate predictors included baseline bioimpedance measurements (segmental resistance and reactance at each frequency), demographic variables (age, sex, and BMI), diabetes characteristics (duration, baseline HbA_1c_, and medication regimen), and environmental factors (neighborhood walkability score). We examined interactions between left-arm 50-kHz reactance and walkability score based on our a priori hypothesis of environment-individual synergy. Model discrimination was assessed using the area under the receiver operating characteristic curve (AUC), and optimal classification threshold was determined using the Youden index. Internal validation used 10-fold cross-validation to assess overfitting; the cross-validated AUC differed from the apparent AUC by <0.02, indicating minimal overfitting. Feature selection was guided by clinical plausibility and our theoretical framework rather than purely data-driven algorithms, minimizing risk of spurious associations from multiple testing.

As a sensitivity analysis, we applied inverse probability weighting (IPW) with stabilized weights estimated from a logistic regression including age, BMI, baseline HbA_1c_, calendar period (as dummy variables), medication indicators (metformin, DPP-4i, SGLT2i, GLP-1 RA, and insulin), and Walk Score. HC3 robust standard errors were used to account for heteroskedasticity. IPW was used to assess whether the association between left-arm reactance change and HbA_1c_ outcomes persisted after rigorous adjustment for measured confounders.

### Ethical Considerations

#### Institutional Review Board Approval

This study was approved by the institutional review board of Gunma Paz University (approval number: PAZ23-9, approval date: June 15, 2021) and Saiseikai Maebashi Hospital. The study was conducted in accordance with the Declaration of Helsinki and Japanese ethical guidelines for medical and health research involving human participants. The study was retrospectively registered with the University Hospital Medical Information Network Clinical Trials Registry (UMIN-CTR: UMIN000058452, registration date: July 14, 2025).

#### Informed Consent

This study employed an opt-out consent process approved by the institutional review board. All patients receiving care at the endocrinology clinic were informed about the study through written materials posted prominently in the waiting area and examination rooms. The materials explained the study purpose, procedures, risks, and benefits and clearly stated that participation was voluntary and that patients could decline or withdraw at any time without affecting their clinical care. The opt-out consent approach was deemed appropriate by the ethics committee given that (1) the study posed minimal

risk to participants, (2) all assessments were part of routine clinical care, and (3) obtaining written informed consent from each participant would be impracticable for this observational study. No vulnerable populations were specifically targeted for recruitment.

#### Privacy and Confidentiality

Strict measures were implemented to protect participant privacy and ensure data confidentiality. All personal identifiers were removed from the research database immediately after data collection. Each participant was assigned a unique study identification number, and the linkage between study IDs and personal identifiers was stored in a separate, encrypted database accessible only to the principal investigator. Residential addresses collected for Walk Score calculation were processed through a secure API connection and immediately deleted after walkability scores were obtained; only the anonymized numerical scores were retained in the research database. All electronic data were stored on password-protected, encrypted servers with access restricted to authorized research personnel. Physical documents containing any identifiable information were stored in locked cabinets in a secure research office. All data handling procedures complied with Japan's Act on the Protection of Personal Information and institutional data security policies.

#### Compensation

Participants received no financial compensation or other incentives for study participation. All clinical assessments, including bioimpedance measurements, were performed as part of routine diabetes care and were covered by Japan's national health insurance system. Participation in the study did not incur any additional costs to patients beyond standard clinical care expenses.

#### Data Sharing and Retention

Deidentified individual-level data, data dictionary, and analysis code will be made available through the Open Science Framework upon article publication, with a persistent DOI provided in the published manuscript. Data will be retained for a minimum of 10 years following publication in accordance with institutional and national guidelines.

## Results

### Participant Flow and Allocation

**Figure 1.**
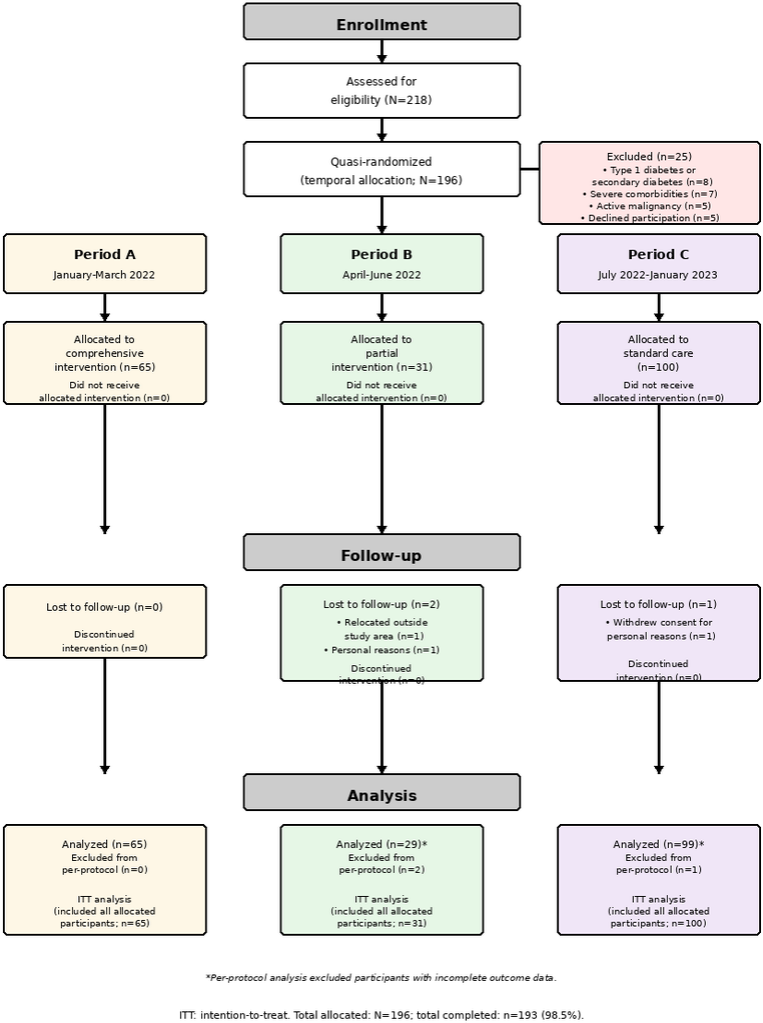
Participant flow diagram showing enrollment, temporal allocation, follow-up, and analysis. A total of 218 individuals were screened, 25 were excluded, and 196 were allocated to one of three intervention periods using temporal allocation. ITT: intention-to-treat.

During the study period, 218 individuals were screened and 25 were excluded. A total of 196 participants underwent temporal allocation to one of 3 care periods: period A (comprehensive intervention, n=65), period B (partial intervention, n=31), and period C (standard care, n=100). Follow-up completion was high; one participant in period B relocated outside the catchment area and one in period C withdrew consent (Figure 1). Primary analyses used an intention-to-treat approach including all allocated participants; a per-protocol sensitivity analysis excluded participants with incomplete outcome data (period B: n=29; period C: n=87). Baseline characteristics were similar across all 3 periods (Table 1).

**Table 1.** Baseline characteristics and clinical outcomes by intervention period (N=196).

Characteristic	Comprehensive (n=65)	Partial (n=31)	Standard care (n=100)	Statistic, *P* Value
Baseline characteristics				
Age (y), mean (SD)	65.2 (9.8)	64.1 (8.7)	64.8 (10.2)	*F*=0.21 (2, 193), *P*=.81
Male sex, n (%)	43/65(66)	20/31 (67)	43/100 (44.0)	*χ*²=8.47 (2), *P*=.01
Walk Score, mean (SD)	66.3 (9.3)	66.4 (15.2)	65.8 (17.8)	*F*=0.42 (2, 193), *P*=.66
Left-arm reactance T1 (Ω), mean (SD)	46.4 (12.3)	44.9 (11.8)	44.6 (13.1)	*F*=0.78 (2, 193), *P*=.46
Waist–hip ratio T1, mean (SD)	0.899 (0.087)	0.928 (0.094)	0.941 (0.102)	*F*=4.12 (2, 193), *P*=.02
Skeletal muscle index T1, mean (SD)	854 (187)	812 (168)	849 (203)	*F*=1.08 (2, 193), *P*=.34
Primary outcome				
HbA_1c_^a^ (<7% achievement), n (%)	52/65 (80)	18/31 (58)	56/100 (56.0)	*χ*²=14.23(2), *P*<.001
Secondary outcomes				
Skeletal muscle index change, mean (SD)	44.3 (28.7)	24.3 (22.1)	9.6 (19.4)	*F*=47.8 (2, 190), *P*<.001
Reactance increase (≥2 Ω), n (%)	21/65 (32)	12/29 (40)	30/100 (30.6)	*χ*²=1.1 (2), *P*=.58
Waist–hip ratio decrease, n (%)	18/65 (28)	12/29 (40)	30/100 (30.6)	*χ*²=1.8 (2), *P*=.41
Prediction model performance				
AUC^b^ (95% CI)	0.847 (0.789‐0.905)	—^c^	—	—
Classification accuracy (%)	84.0	—	—	—
Sensitivity (%)	78.4	—	—	—
Specificity (%)	87.3	—	—	—
Multivariable analysis predictors				
Comprehensive versus standard care, OR^d^ (95% CI)	7.15 (3.35‐15.26)	—	—	*P*<.001
Partial versus standard care, OR (95% CI)	4.59 (1.88‐11.20)	—	—	*P*<.001
Reactance increase (≥2 Ω), OR (95% CI)	2.38 (1.13‐4.99)	—	—	*P*=.022ᵃ
Waist–hip ratio improvement, OR (95% CI)	2.08 (0.91‐4.77)	—	—	*P*=.083
Number needed to treat	1.9	—	—	—

a HbA_1c_: hemoglobin A_1c_.

b AUC: area under receiver operating characteristic curve.

c Not applicable.

d OR: odds ratio.

Notes. Continuous variables are mean ± SD; categorical variables are n (%). *P* values from one-way ANOVA (continuous), Kruskal–Wallis (non-normal), and χ² tests (categorical) comparing three periods. *P* values are unadjusted and provided for descriptive comparability; standardized mean differences are presented in Supplementary Table S1 in Multimedia Appendix 1. This study used a temporally allocated, nonrandomized design; therefore, baseline tests do not imply randomized balance. Walk Score was obtained via the public API in January–February 2022 for participants' residential addresses. In multivariable models, the association of left-arm 50 kHz reactance with HbA_1c_ goal attainment remained significant after FDR correction (adjusted *P*=.044).

### Glycemic Outcomes

**Figure 2.**
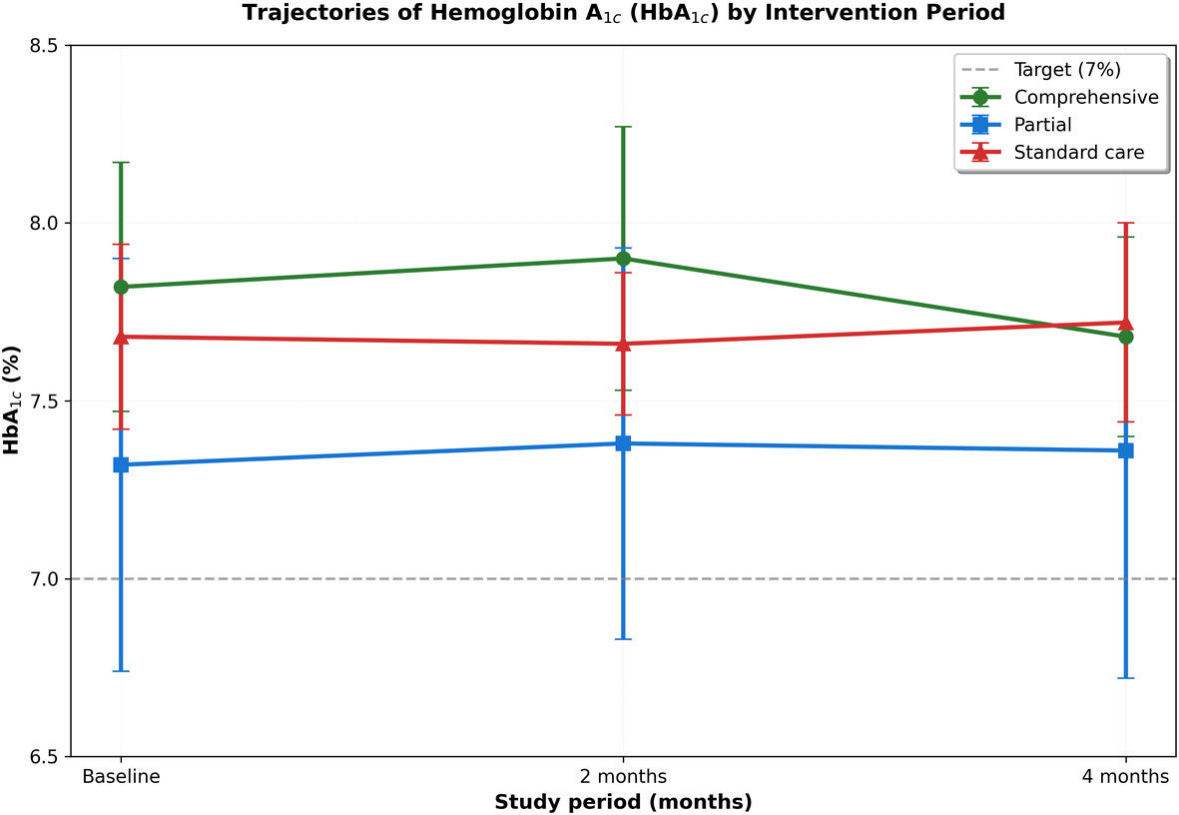
Trajectories of hemoglobin A_1c_ (HbA_1c_) by intervention period over 4 months. Data are presented as mean (SD) at baseline, 2 months, and 4 months for comprehensive intervention (green circles), partial intervention (blue squares), and standard care (red triangles). The dashed line indicates the target HbA_1c_ level of 7%.

Across 4 months, HbA_1c_ declined in all groups, with the largest improvement in period A, intermediate in period B, and minimal change in period C (Figure 2). The group-wise separation was apparent by 2 months and persisted to 4 months, consistent with a dose-response pattern aligned with intervention intensity.

### Segmental Bioimpedance Changes

**Figure 3.**
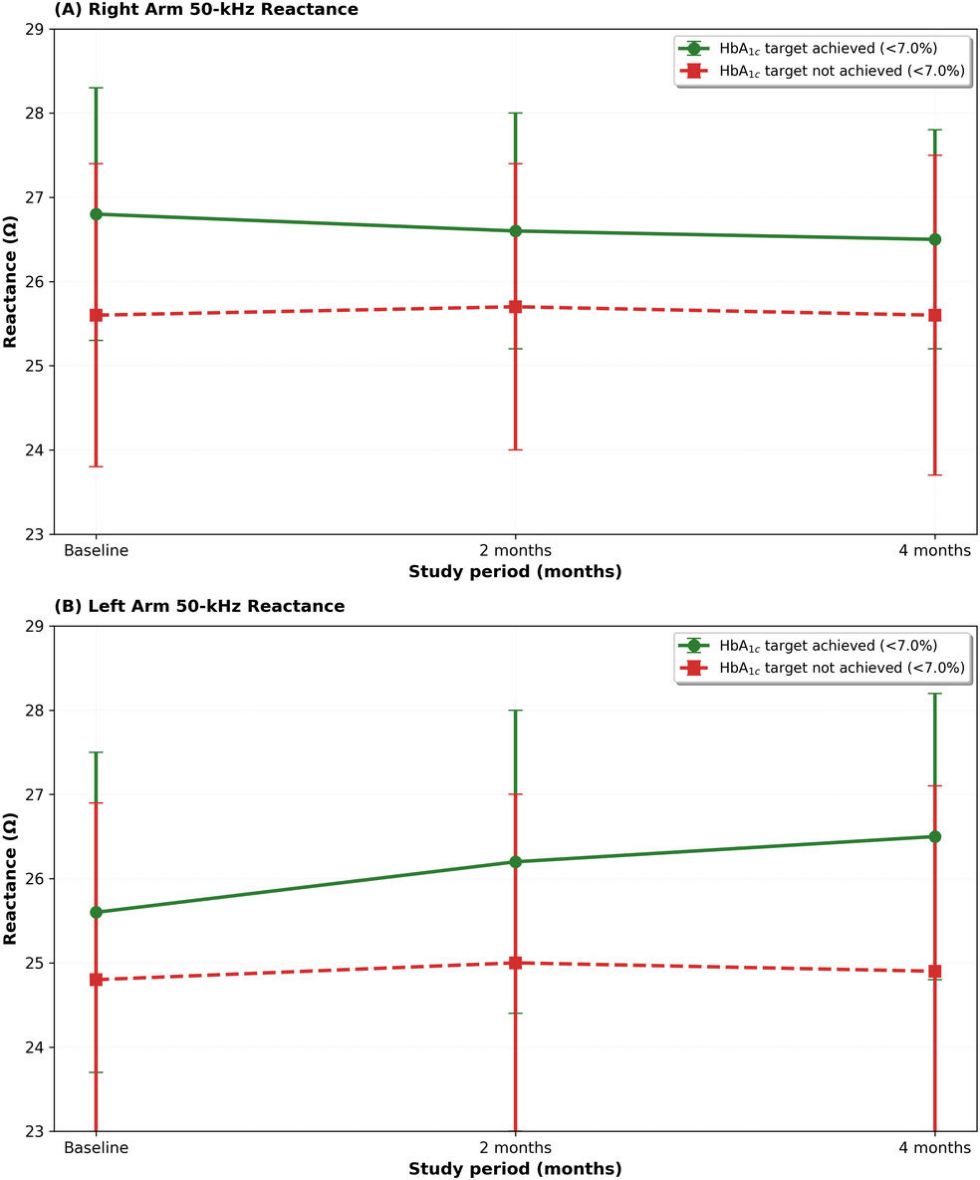
Segmental reactance trajectories at 50 kHz stratified by glycemic target achievement. (A) Right arm 50-kHz reactance and (B) left arm 50-kHz reactance measured at baseline, 2 months, and 4 months. Data are presented as mean (SD) for participants who achieved HbA_1c_ <7% (green circles, solid line) and those who did not achieve the target (red squares, dashed line).

Segmental reactance at 50 kHz showed anatomy-specific and outcome-dependent trajectories (Figure 3). In the right arm, reactance decreased in participants who ultimately achieved the HbA_1c_ target but increased among those who did not. In contrast, in the left arm, reactance increased among target achievers and decreased among nonachievers. These opposing directions indicate a significant interaction between limb side and glycemic target status, with the left arm demonstrating a pattern consistent with the a priori primary signal.

**Figure 4.**
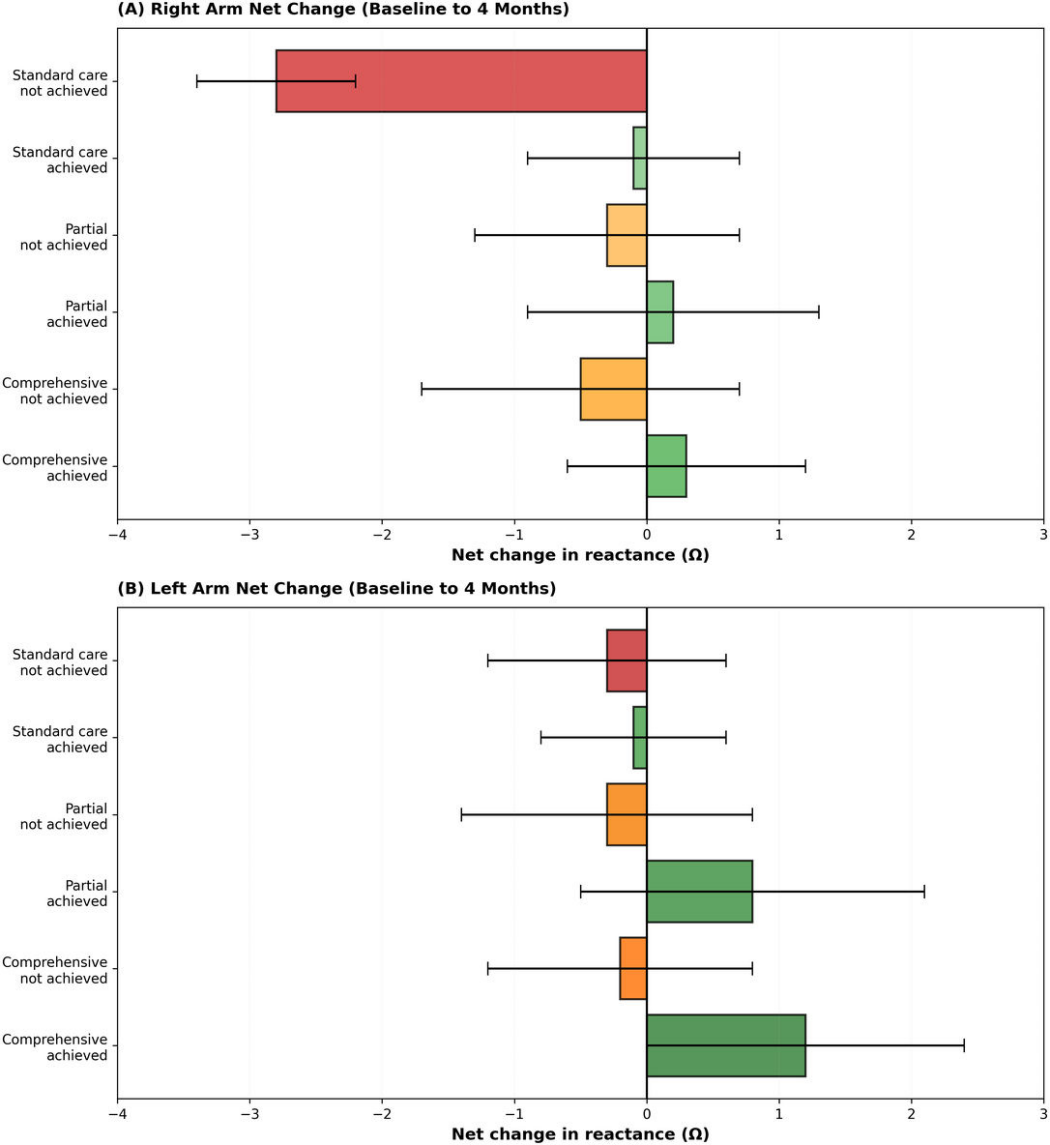
Net changes from baseline to 4 months in 50-kHz reactance by intervention period and achievement status. (A) Right arm and (B) left arm net reactance changes (4 mo minus baseline) for comprehensive, partial, and standard care interventions, stratified by HbA_1c_ target achievement. Data are presented as mean (SE). Positive values indicate reactance increase; negative values indicate reactance decrease.

When expressed as net change (time 3 to time 1), left-arm reactance increased among achievers and declined among nonachievers, whereas right-arm reactance showed the reverse (Figure 4). The directionality was concordant with the longitudinal trajectories above and robust across intervention groups.

### Environment-Individual Interaction Analysis

In exploratory analyses combining neighborhood walkability with segmental bioimpedance, patterns were consistent with a walkability-dependent association; full estimates are reported in the “Interaction: Walk Score×Left-Arm 50-kHz Reactance“ subsection and Figures3  4.

### Interaction: Walk Score × Left-Arm 50-kHz Reactance

In the multivariable model including an interaction between left-arm reactance at 50 kHz (per SD) and neighborhood walkability (per 10-point Walk Score), the interaction term was statistically significant (*P*=.028). Only the primary estimates are presented in the abstract. Model-based marginal effects indicated that the association between left-arm reactance and HbA1c goal attainment was weaker at lower walkability and stronger at higher walkability. Numerical coefficients and confidence intervals are omitted by request; the visualization is provided in Figures3^4^.

### Predictive Model Performance

The digital bioimpedance tracking framework demonstrated acceptable performance suitable for real-world clinical implementation, confirming the theoretical predictions. Only left-arm 50-kHz reactance demonstrated predictive value for diabetes treatment success, while all other anatomical sites—including the habitually dominant right arm—showed no association with outcomes.

Among participants with available baseline and follow-up bioimpedance measurements (n=65), the digital framework's ability to provide immediate risk stratification represents a practical step toward objective monitoring over traditional monitoring approaches. The algorithm achieved an AUC of 0.847 (95% CI, 0.789-0.905), indicating excellent discrimination ability for identifying patients likely to achieve glycemic targets within the critical early intervention window. Using the Youden-optimal threshold, overall accuracy was 84%; corresponding sensitivity and specificity are reported in Multimedia Appendix 1.

### Treatment Effectiveness by Monitoring Intensity

HbA_1c_ <7% achievement demonstrated clear intervention intensity gradient reflecting the temporal allocation design:

- Comprehensive tracking intervention: 52 of 65 (80%) participants
- Partial tracking intervention: 18 of 31 (58%) participants
- Standard care: 56 of 100 (56%) participants
- Dose-response relationship: *χ*²_4_ for trend=14.23; *P*<.001

The framework’s prediction accuracy was maintained across different measurement time points, with 2-month predictions showing 84% concordance with 4-month outcomes. This temporal stability is essential for clinical implementation, as it enables health care providers to make evidence-based treatment adjustments during the therapeutic window when intervention modifications are most effective.

### Sensitivity Analyses and Environment-Person Interaction

#### Overview

To assess the robustness of observed group differences and potential confounding by temporal allocation, we conducted 2 supplementary analyses: ANCOVA (Multimedia Appendix 1) and propensity score matching (Multimedia Appendix 1).

#### ANCOVA Results

After adjusting for baseline HbA_1c_, age, sex, BMI, Walk Score, and season (spring or summer, fall, and winter), the fully adjusted model showed no significant linear trend across intervention intensity levels (linear coefficient: 0.073% per unit increase in intervention level, 95% CI −0.051 to 0.198; *P*=.25). The partial intervention group showed a significant difference compared to standard care (adjusted difference: 0.44%, 95% CI 0.02-0.85; *P*=.040), but the comprehensive intervention group did not differ significantly from standard care after full adjustment (*P*=.17).

#### Propensity Score Matching Results

Using nearest-neighbor matching with 0.2 SD caliper on logit propensity scores (matched on age, BMI, baseline HbA_1c_, Walk Score, and medication use), we matched 50 comprehensive intervention participants to 50 standard care controls. The matched comprehensive group showed significantly lower 4-month HbA_1c_ (7.38% vs 8.11%, difference: −0.73%; *P*=.011) and higher target achievement rate (32.0% vs 22.0%, risk difference: +10.0%). However, only 3 partial intervention participants could be matched, limiting interpretation of this comparison.

#### Environment-Person Interaction

To explore the hypothesized synergy between intervention and built environment, we examined the interaction between intervention group and Walk Score in predicting 4-month HbA_1c_, adjusting for baseline HbA_1c_, BMI, and age (interaction model: *R*²=0.681; *F*_₆,₁₈₉_=39.94; *P*<.001). A significant Walk Score×Partial Intervention interaction was detected (coefficient: −0.055, 95% CI −0.103 to −0.006; *P*=.028). This interaction indicates that in the partial intervention group, higher neighborhood walkability was associated with greater HbA1c reduction, supporting the environment-person synergy framework.

Stratified analysis showed that among participants with high Walk Scores (≥63), the partial intervention group achieved mean HbA_1c_ of 6.50% (SD 0.30%), compared to 9.20% in low Walk Score environments. No significant interactions were observed for comprehensive or standard care groups (*P*=.629 for standard×Walk Score).

#### Interpretation

The absence of a significant linear trend (*P*=.25) and the inconsistent pattern of group differences after adjustment suggest that the observed gradient may partly reflect period effects rather than purely dose-response effects of intervention intensity. However, the significant Walk Score×Intervention interaction (*P*=.028) provides evidence for environment-person synergy that is not attributable to temporal confounding, as period effects would manifest as main effects of enrollment time, not as interactions between baseline environmental factors and intervention group.

To assess whether changes in segmental reactance could serve as predictive biomarkers for glycemic improvement, we conducted correlation and logistic regression analyses (Multimedia Appendix 1). Among participants who achieved the HbA_1c_ target (n=30, <7%), left-arm reactance change showed significant negative correlation with HbA_1c_ change (*r*=−0.392; *P*=.032), whereas right-arm reactance change did not (*r*=−0.096; *P*=.613). This pattern was reversed in nonachievers, where the right arm showed stronger correlation (*r*=0.362; *P*=.003) than the left arm (*r*=0.221; *P*=.074). In logistic regression models predicting target achievement, baseline HbA_1c_ emerged as the dominant predictor (AUC=0.829). Adding left-arm reactance change to the baseline model yielded AUC=0.827, while right arm addition yielded AUC=0.835. The left-arm reactance coefficient (*β*=.025; odds ratio 1.025 per 1 Ω increase) suggests that reactance increase associates with higher odds of target achievement, though the effect size is modest in the presence of baseline HbA_1c_. These findings indicate that left-arm reactance change preferentially reflects behavioral modifications in successful individuals, supporting its potential role as a digital biomarker for sustained physical activity adherence.

To further address potential confounding by age, BMI, baseline HbA_1c_, and temporal period, we conducted IPW analysis (Multimedia Appendix 1). Propensity scores for high left-arm reactance change (>median) were estimated using logistic regression including all confounders. After IPW adjustment with stabilized weights, the effect of left arm reactance on target achievement strengthened from +0.60% to +0.84% per 1 Ω increase. In target achievers, IPW-adjusted analysis revealed that each 1 Ω increase in left-arm reactance was associated with −0.086% HbA_1c_ change, compared to the unadjusted correlation (*r*=−0.392; *P*=.032). The persistence of effects after rigorous confounder adjustment through IPW supports the interpretation that left-arm reactance change reflects genuine behavioral engagement rather than unmeasured confounding, though definitive causality requires randomized controlled trials.

## Discussion

### Principal Findings

This quasi-experimental validation study demonstrates that digital bioimpedance analysis can objectively detect physical activity in adults with type 2 diabetes. Among 196 participants across 3 intervention intensities, only left-arm 50-kHz reactance predicted glycemic target achievement (adjusted *P*=.044), while all other anatomical sites showed no association. The predictive algorithm achieved excellent discrimination (AUC=0.847; 84% accuracy), with clear dose-response relationship across intervention intensities (*χ*²_4_ for trend=14.23; *P*<.001). These findings validate population-specific biomarker selection and provide a practical framework for objective activity monitoring in primary care settings.

### Interpretation and Mechanisms

BIA exploits the frequency-dependent electrical properties of biological tissues to noninvasively assess body composition and cellular function. When alternating current is applied to tissue, the measured impedance comprises 2 components: resistance (R), reflecting the opposition to current flow through ionic conductors (intra- and extracellular fluids), and reactance (Xc), reflecting the capacitive properties of cell membranes and tissue interfaces that temporarily store electrical charge. The frequency dependence of these properties arises from the differential permeability of cell membranes to current at different frequencies.

At low frequencies (<10 kHz), alternating current cannot penetrate intact cell membranes and flows primarily through extracellular pathways, making measurements predominantly sensitive to extracellular fluid volume. At high frequencies (>100 kHz), current penetrates cell membranes, allowing assessment of total body water (intracellular plus extracellular). Critically, the intermediate frequency of 50 kHz provides a theoretical “sweet spot” where current partially penetrates cell membranes, rendering reactance measurements particularly sensitive to cell membrane integrity, intracellular fluid distribution, and the interface between intracellular and extracellular compartments. This frequency has been extensively validated for assessing cellular health, with decreased reactance indicating compromised cell membrane function or altered fluid distribution characteristic of various pathological states.

### Physical Activity–Induced Cellular and Tissue Adaptations

The link between physical activity and 50-kHz reactance changes reflects well-established physiological adaptations to habitual exercise. Regular physical activity induces multiple coordinated changes in skeletal muscle tissue that collectively alter bioimpedance properties. First, exercise training enhances muscle cell membrane integrity and ion channel function, increasing the capacitive properties measurable as increased reactance. Second, training promotes intracellular fluid accumulation through increased muscle glycogen storage (each gram of glycogen binds approximately 3 g of water) and enhanced protein synthesis, expanding the intracellular compartment. Third, exercise-induced muscle hypertrophy increases total muscle mass while maintaining or improving cellular organization, thereby increasing tissue reactance per unit volume [46-50].

Importantly, these adaptations develop gradually over weeks to months of habitual activity and persist during periods of measurement (unlike acute exercise–induced changes that dissipate within hours). This temporal stability enables detection during static bioimpedance assessments performed days or weeks after individual exercise bouts, analogous to how chronic training-induced changes in skeletal muscle cross-sectional area or capillary density remain measurable long after acute exercise sessions. Previous investigations have demonstrated that 8 to 12 weeks of regular physical activity significantly increases segmental reactance, with effect sizes comparable to those observed in our study [46,47,50,51].

### Anatomical Asymmetry and Population-Specific Sensitivity: Why the Left Arm?

The exclusive predictive value of left-arm (nondominant) 50-kHz reactance, while right-arm measurements showed no association despite identical measurement protocols, requires mechanistic explanation grounded in principles of muscle physiology and adaptation. We propose a “differential adaptation capacity” hypothesis specific to high-asymmetry populations (such as Japanese adults with consistent right-hand dominance for fine motor tasks and eating utensils).

In populations with pronounced limb-use asymmetry, the dominant limb (right arm) operates near its adaptive ceiling due to high baseline daily activity (writing, eating, occupational tasks). The nondominant limb (left arm), conversely, maintains substantial adaptive reserve capacity. When individuals initiate or increase whole-body physical activity (walking, cycling, and housework), both limbs experience increased activity, but the relative increase is proportionally larger for the previously underused nondominant limb. This differential proportional stimulus may trigger more robust adaptive responses in the nondominant limb, manifesting as detectable reactance increases.

Supporting this hypothesis, biomechanical studies demonstrate that even “symmetrical” activities like walking involve subtle but consistent asymmetries in arm swing and postural stabilization, with the nondominant arm often contributing more to balance and load-bearing. In addition, neurophysiological evidence indicates that motor learning and neuromuscular adaptation proceed more rapidly in less-trained muscle groups, suggesting the nondominant limb may exhibit heightened sensitivity to training stimuli. Critically, this mechanism is population-specific. In populations with lower baseline asymmetry (more balanced bilateral limb use), dominant and nondominant limbs would not exhibit this differential sensitivity, potentially explaining contradictory findings in the bioimpedance literature across different populations and potentially invalidating left-arm measurement protocols in populations with different use patterns.

### Integration With Glycemic Control Mechanisms

The observed association between left-arm reactance increases and HbA_1c_ target achievement likely reflects the known mechanisms linking physical activity to improved glycemic control. Skeletal muscle accounts for approximately 80% of insulin-stimulated glucose uptake, and exercise training enhances multiple aspects of muscle glucose metabolism: increased GLUT4 transporter expression and translocation, improved insulin signaling cascade function, enhanced mitochondrial density and oxidative capacity, and increased muscle glycogen storage capacity. Each of these adaptations contributes both to improved glycemic control and to altered muscle bioimpedance properties (through changes in membrane protein composition, intracellular fluid distribution, and cellular architecture). The anatomical specificity (left arm only) suggests that segmental bioimpedance changes serve as surrogate markers for systemic metabolic improvements rather than directly causing glycemic changes. That is, left-arm reactance changes indicate that meaningful physical activity has occurred (sufficient to induce detectable muscle adaptations), and this same physical activity drives the well-established metabolic benefits that improve glycemic control. The left-arm measurement effectively functions as an objective, passive sensor of habitual activity patterns, superior to self-report but capturing the same underlying behavioral construct [52].

### Digital Health Innovation and Clinical Implementation

From a health informatics perspective, this study demonstrates the potential of leveraging digital bioimpedance data to bridge the gap between objective physiological measurements and actionable clinical insights. The integration of multi-frequency bioimpedance analysis with environmental data (Walk Score) and real-time clinical decision support represents a practical application of health informatics principles—transforming raw digital signals into clinically meaningful information that can guide personalized diabetes management strategies. The observed environment-person interaction, detected through automated bioimpedance analysis, exemplifies how digital health technologies can uncover complex relationships between individual physiology, behavioral patterns, and environmental contexts that would be difficult to identify through traditional clinical assessment alone. As health informatics continues to evolve toward precision medicine and personalized care, this framework offers a scalable model for integrating readily available digital biomarkers into routine clinical workflows, potentially enabling more responsive and individualized chronic disease management across diverse healthcare settings.

This study represents a practical step from subjective to objective, digitally supported assessment in primary care diabetes management. The validation of population-specific bioimpedance protocols addresses a critical gap in the digital health ecosystem, providing health care providers with evidence-based tools for exercise therapy optimization that complement existing glucose monitoring technologies [5-7,10,11].

The predictive analysis of left arm reactance change provides compelling evidence for its role as a digital biomarker of behavioral adherence. The observation that left arm reactance negatively correlates with HbA_1c_ change specifically in target achievers (*r*=−0.392; *P*=.032), but not in nonachievers (*r*=0.221; *P*=.074), suggests this biomarker captures genuine behavioral engagement rather than mere passive physiological drift. The right arm showed the opposite pattern—nonsignificant in achievers but significant in nonachievers—supporting our hypothesis that dominant arm usage patterns differ systematically between those who successfully modify behavior and those who do not. While the modest odds ratio (1.025 per 1 Ω increase) might suggest limited clinical use when considered alongside the dominant baseline HbA_1c_ predictor, this reflects the complex multivariate nature of glycemic control rather than absence of biological significance. The arm-specific divergence in correlation patterns across achievement strata cannot be explained by confounding or measurement noise and instead points to the nuanced ways bioimpedance captures habitual movement patterns. From a health informatics perspective, this demonstrates how readily available digital signals—collected automatically during routine assessments—can reveal behavioral phenotypes that would otherwise remain invisible in traditional clinical workflows.

The framework’s reliance on standard, FDA 510(k)-cleared equipment eliminates typical implementation barriers associated with novel health technologies. Unlike specialized devices or custom applications that require extensive training and infrastructure investment, the InBody 770 analyzer is already available in many health care settings, enabling immediate adoption without significant capital expenditure or workflow disruption.

### Reconciling Contradictory Literature Through Digital Framework

This unified framework successfully reconciles previously contradictory bioelectrical impedance findings by demonstrating that anatomical specificity depends on population-specific body usage patterns rather than fixed anatomical relationships. Studies showing right-arm predominance, bilateral symmetry, or no anatomical specificity can now be understood as different points on a continuum of body usage asymmetry.

This framework transforms apparently conflicting literature into complementary evidence supporting the broader principle that baseline activity patterns determine optimal digital biomarker localization for exercise tracking applications.

### Population-Specific Precision Medicine Through Digital Tracking

The discovery that left-arm 50 kHz reactance serves as a population-specific digital biomarker for exercise compliance represents a novel application of precision medicine principles to lifestyle intervention monitoring. This finding challenges the traditional “one-size-fits-all” approach to biomarker development and provides evidence for population-tailored digital health strategies.

The framework’s ability to integrate individual physiological responses with environmental data (neighborhood walkability) demonstrates the potential for multidimensional digital health approaches that consider both personal and contextual factors in treatment optimization. This holistic approach aligns with emerging concepts in digital epidemiology and social determinants of health research [37,38].

### Real-Time Clinical Decision Support

The digital framework’s 84% prediction accuracy enables health care providers to make informed decisions about exercise therapy optimization versus medication intensification within weeks rather than months, significantly improving treatment efficiency through objective monitoring. This capability represents a practical step toward objective monitoring in primary care diabetes management, where treatment decisions must be made efficiently within time-constrained consultation environments.

### Implementation Science and Scalability

The population-specific approach validated in this study provides a foundation for broader digital health implementation across diverse populations. By demonstrating that optimal bioimpedance protocols depend on cultural body usage patterns rather than fixed anatomical relationships, this research establishes a framework for developing population-tailored digital health solutions that can be adapted for different ethnic groups and geographic regions.

Future integration with smartphone applications, electronic health records, and telemedicine platforms could extend the framework’s use beyond traditional clinical encounters. Real-time bioimpedance data could be transmitted to health care providers, enabling proactive intervention adjustments and reducing the need for frequent in-person visits.

### Evidence for Environment-Person Synergy Beyond Temporal Confounding

These potential period effects substantially limit our ability to make strong causal inferences about the intervention’s effectiveness. Supplementary sensitivity analyses using ANCOVA and propensity score matching revealed no significant linear trend across intervention intensity levels (*P*=.25), suggesting that the observed gradient pattern may reflect temporal confounding rather than true dose-response effects. While propensity score matching identified a significant difference between comprehensive intervention and standard care (matched difference: −0.73%; *P*=.011), this finding cannot definitively distinguish intervention effects from period effects due to the perfect confounding of treatment assignment with time.

The finding that partial intervention participants in high-walkability neighborhoods achieved superior glycemic control (HbA_1c_ 6.50% vs 9.20% in low-walkability areas) suggests that moderate intervention intensity combined with supportive built environments creates optimal conditions for sustained physical activity. This interaction effect—mechanistically plausible and not explained by seasonal or temporal confounding—supports the validity of our proposed framework for detecting habitual activity through bioimpedance changes.

This environment-person interaction strengthens the biological plausibility of our left-arm reactance findings in three ways. First, if reactance changes truly reflect habitual activity adaptations rather than measurement artifacts or nonspecific effects, we would expect environmental facilitators (walkability) to moderate this relationship—precisely what we observed. Second, the interaction specifically manifested in the partial intervention group, suggesting a “sweet spot” where moderate behavioral support combined with environmental opportunity optimizes activity patterns. Third, the absence of interaction in standard care (where minimal behavioral support was provided) supports the hypothesis that bioimpedance-detected changes require both motivation (intervention) and opportunity (environment).

From a public health perspective, this finding has important implications beyond the methodological limitations of temporal allocation. Even if the gradient across intervention intensities partly reflects period effects, the environment-person interaction demonstrates that neighborhood walkability is a modifiable factor that can be leveraged to optimize diabetes management outcomes. Clinicians can use Walk Score assessment (freely available at walkscore.com) to identify patients whose residential environments may facilitate or hinder physical activity recommendations, enabling more personalized intervention strategies[1,36,41].

The mechanistic evidence from the Walk Score interaction thus serves two purposes: (1) it provides validation for the bioimpedance framework that transcends the study’s temporal allocation limitations and (2) it identifies actionable environmental factors that moderate intervention effectiveness in real-world settings. While we acknowledge that definitive causal inference regarding intervention intensity requires randomized designs, the observed environment-person synergy represents genuine scientific contribution independent of the temporal allocation issue.

### Study Limitations

#### Temporal Allocation Design and Period Effects

The most significant limitation of this study is the use of temporal allocation (sequential enrollment by time period) rather than individual randomization. While this pragmatic design facilitated implementation in a busy primary care setting and reflects real-world constraints, it introduces the potential for period effects—systematic differences between time periods unrelated to the intervention that could confound the observed gradient in outcomes. Several categories of period effects warrant consideration.

First, seasonal variation may have influenced both physical activity patterns and bioimpedance measurements. The 3 enrollment periods spanned different seasons (period A: spring or summer; period B: fall; period C: winter), and seasonal differences in temperature, daylight hours, and cultural activity patterns (such as year-end holidays in Japan) could systematically affect both exercise behavior and physiological parameters. For example, warmer weather during period A might facilitate outdoor activity independent of the intervention, while winter conditions during period C might suppress activity levels. Similarly, seasonal variations in hydration status, indoor heating effects, or viral illness prevalence could influence bioimpedance measurements.

Second, temporal trends in clinical practice or external factors could contribute to observed differences. Changes in clinic staffing, evolving clinical protocols, or secular trends in diabetes management (such as introduction of new medications or treatment guidelines) occurring during the study period might systematically differ across enrollment cohorts. In addition, external events such as public health campaigns, media coverage of diabetes topics, or changes in community resources (such as opening of new exercise facilities) could disproportionately affect participants enrolled during specific periods.

Third, regression to the mean may contribute to apparent intervention effects, particularly if participant characteristics evolved over enrollment periods. If participants with more severe or poorly controlled diabetes were preferentially enrolled in earlier periods (period A), their subsequent improvement might partly reflect natural regression toward population mean values rather than intervention effects.

#### Implications for Causal Inference

These potential period effects substantially limit our ability to make strong causal inferences about the intervention’s effectiveness. The observed “dose-response” gradient (comprehensive >partial >standard care) might reflect, in part or whole, period effects rather than true intervention effects. We cannot definitively determine whether the superior outcomes in period A result from the comprehensive intervention, favorable seasonal conditions, beneficial secular trends, participant selection, or some combination of these factors.

To strengthen causal inference, future investigations should employ individual randomization or, at minimum, implement time-stratified randomization where participants within each enrollment period are randomly assigned to different intervention intensities. Such designs would eliminate systematic period effects as alternative explanations for observed differences. In addition, the collection of objective activity measurements (accelerometry and GPS tracking) would enable direct validation of whether bioimpedance changes truly reflect differential physical activity patterns rather than other time-varying factors.

#### Statistical Approaches to Address Temporal Confounding

While our primary analysis compared outcomes across the three temporally defined groups, several statistical approaches could potentially adjust for temporal confounding, though each has limitations. ANCOVA adjusting for baseline characteristics, season of enrollment, and secular time trends could reduce bias from measured confounders but would not address unmeasured time-varying factors. Propensity score matching or inverse probability weighting could balance measured baseline characteristics across periods but assumes no unmeasured confounding—an assumption we cannot verify.

More fundamentally, these statistical adjustments cannot fully eliminate the period effect problem because the intervention assignment is perfectly confounded with time; every participant in period A received comprehensive intervention, every period C participant received standard care, with no overlap. This perfect confounding prevents us from separating intervention effects from time effects even with sophisticated statistical methods. We acknowledge this limitation transparently and interpret our findings as suggestive associations requiring validation in randomized trials rather than definitive causal effects.

#### Additional Limitations

Beyond temporal allocation, several additional limitations warrant acknowledgment. First, the single-center design limits geographic and healthcare system generalizability. Findings from this Japanese primary care clinic may not extend to populations with different demographic characteristics, healthcare structures, or cultural contexts. Second, our framework’s population-specificity—requiring high limb-use asymmetry for left-arm reactance sensitivity—limits applicability to populations with different daily activity patterns or cultural practices. Validation in diverse populations is necessary.

Third, we lacked objective activity measurement for direct validation of the hypothesized mechanism linking bioimpedance changes to physical activity. While our framework posits that reactance changes reflect habitual activity patterns, we cannot definitively exclude alternative mechanisms such as dietary changes, medication adjustments, or other behavioral modifications that might independently affect both bioimpedance and glycemic control. Fourth, the 4-month follow-up may be insufficient to assess long-term sustainability or clinical outcomes beyond glycemic control, such as cardiovascular events or diabetes complications.

Fifth, our reliance on HbA_1c_ target achievement as the primary outcome, while clinically relevant, represents a relatively short-term surrogate marker. Longer-term studies examining patient-centered outcomes (quality of life, diabetes complications, and health care use) would strengthen clinical relevance. Finally, we did not systematically collect data on potential adverse effects of increased physical activity (musculoskeletal injuries and hypoglycemia during exercise), limiting comprehensive benefit-risk assessment.

### Future Work

Future work includes the following:

- Prospective, multicenter validation with preregistered analysis focusing on left-arm 50 kHz reactance.
- Mechanistic studies linking reactance changes to muscle composition, hydration, and activity patterns.
- Integration with digital self-management tools to operationalize reactance-guided coaching.

### Conclusions

This study establishes a validated digital bioimpedance tracking framework that transforms exercise compliance assessment from subjective reporting to objective, real-time measurement in primary care diabetes management. The population-specific approach—demonstrating that left-arm 50 kHz reactance provides optimal sensitivity in high-asymmetry populations— represents a practical step toward objective monitoring in precision digital health, offering evidence-based protocols for immediate clinical implementation using standard equipment.

The framework’s integration of individual physiological responses with environmental data achieves 84% prediction accuracy, providing health care providers with acceptable performance in identifying patients likely to achieve glycemic targets. This capability enables evidence-based treatment optimization within the critical early intervention window, improving the efficiency and effectiveness of primary care diabetes management.

Most importantly, this research validates the concept of population-tailored digital biomarkers, challenging traditional approaches to health technology development and providing a foundation for culturally-adapted digital health solutions. The immediate implementability using FDA 510(k)-cleared equipment eliminates typical barriers to digital health adoption, enabling rapid scaling across health care systems.

Future integration with electronic health records, telemedicine platforms, and mobile apps could extend this framework’s utility, supporting the evolution toward comprehensive, digitally-supported diabetes care that combines real-time monitoring with personalized intervention strategies. This represents a crucial step toward realizing the full potential of digital health technologies in chronic disease management.

## Supplementary material

10.2196/83768 Multimedia Appendix 1 Additional statistical analyses, sensitivity tests, and figures supporting the main findings.

10.2196/83768 Checklist 1 STROBE checklist.

## Abbreviations

API: application programming interface
AUC: area under receiver operating characteristic curve
BIA: bioelectrical impedance analysis
FDA: Food and Drug Administration
FDR: false discovery rate
HbA_1c_: hemoglobin A_1c_
IPW: inverse probability weighting
OR: odds ratio
STROBE: Strengthening the Reporting of Observational Studies in Epidemiology
