# Supplementary material for: Digital Bioimpedance for Physical Activity Detection in Type-2 Diabetes: Quasi-Experimental Validation Study
Source: JMIR Diabetes. 2025 Dec 16;10:e83768. doi: 10.2196/83768 (PMC12707439; doi:10.2196/83768)
Supplement: Multimedia Appendix 1 [file diabetes-v10-e83768-s001.docx]

**Supplementary Materials**

Digital Bioimpedance for Physical Activity Detection in Type 2 Diabetes: Quasi-Experimental Validation Study

*JMIR Diabetes 2025 (Manuscript ID: 83768)*

# Supplementary Table S1

*ANCOVA Results with Full Covariate Adjustment*

| **Model** | **Coefficient (95% CI)** | **P-value** |
| --- | --- | --- |
| Linear trend (per intervention level) | 0.073 (-0.051 to 0.198) | 0.25 |
| Partial vs Standard care | 0.44 (0.02 to 0.85) | 0.040 |
| Comprehensive vs Standard care | (Not significant after adjustment) | 0.17 |

Note: Analysis of covariance examining 4-month HbA1c across intervention periods, adjusting for baseline HbA1c, age, sex, BMI, Walk Score, and season. The absence of significant linear trend (P=0.25) suggests potential temporal confounding.

# Supplementary Table S2

*Propensity Score Matching Analysis*

| **Comparison** | **Mean Difference (95% CI)** | **P-value** |
| --- | --- | --- |
| 4-month HbA1c (%) | -0.73 (Comprehensive vs Standard) | 0.011 |
| Target achievement (%) | +10.0% (32.0% vs 22.0%) | <0.05 |
| Matched pairs | 50 per group | — |

Note: Results from nearest-neighbor matching (0.2 SD caliper) comparing comprehensive intervention to standard care. Matched on age, BMI, baseline HbA1c, Walk Score, and medication use. Only 3 partial intervention participants could be matched, limiting interpretation of this comparison.

# Supplementary Table S2c

*Walk Score × Intervention Interaction Model*

| **Term** | **Coefficient (95% CI)** | **P-value** |
| --- | --- | --- |
| Walk Score × Partial Intervention | -0.055 (-0.103 to -0.006) | 0.028 |
| Walk Score × Comprehensive Intervention | (Not significant) | 0.629 |
| Model R² | 0.681 | <0.001 |

Note: Full multivariable regression model testing environment-person synergy (R²=0.681, F=39.94, P<0.001). The significant Walk Score × Partial Intervention interaction (P=0.028) indicates that higher neighborhood walkability enhances partial intervention effectiveness. Stratified analysis showed partial intervention participants in high-walkability areas (Walk Score ≥63) achieved mean HbA1c of 6.50±0.30% compared to 9.20% in low-walkability environments.

# Supplementary Table S4

*Predictive Analysis of Left Arm Reactance*

| **Predictor / Outcome** | **Correlation / OR (95% CI)** | **P-value** |
| --- | --- | --- |
| Left-arm reactance (achievers) | r = -0.392 | 0.032 |
| Right-arm reactance (achievers) | r = -0.096 | 0.613 |
| Left-arm reactance (non-achievers) | r = 0.221 | 0.074 |
| Right-arm reactance (non-achievers) | r = 0.362 | 0.003 |
| Baseline HbA1c (predictor) | AUC = 0.829 | — |
| Model with left-arm reactance | AUC = 0.827 | — |

Note: Correlation analyses between segmental reactance changes and HbA1c outcomes, stratified by target achievement status. Left-arm reactance shows divergent patterns between achievers (negative correlation) and non-achievers (weak positive), while right-arm shows the opposite pattern. This arm-specific divergence supports the hypothesis that left-arm reactance captures genuine behavioral engagement.

# Supplementary Table S5

*Inverse Probability Weighting (IPW) Sensitivity Analysis*

| **Analysis** | **Effect Estimate** | **Interpretation** |
| --- | --- | --- |
| IPW-adjusted effect (per 1Ω) | +0.84% vs +0.60% (unadjusted) | Strengthened after adjustment |
| Target achievers (IPW) | -0.086% HbA1c per 1Ω | Significant negative association |
| Persistence after adjustment | Effect maintained | Supports behavioral mechanism |

Note: IPW-adjusted estimates examining the association between left-arm reactance and glycemic outcomes after rigorous confounder adjustment. Propensity scores for high left-arm reactance change (>median) were estimated using logistic regression including age, BMI, baseline HbA1c, calendar period, medication indicators, and Walk Score. The strengthened effect after adjustment (from +0.60% to +0.84% per 1Ω) supports a genuine behavioral mechanism rather than unmeasured confounding.

# Supplementary Figure S4

*ROC Curve for Left Arm 50-kHz Reactance Predicting HbA1c Target Achievement*


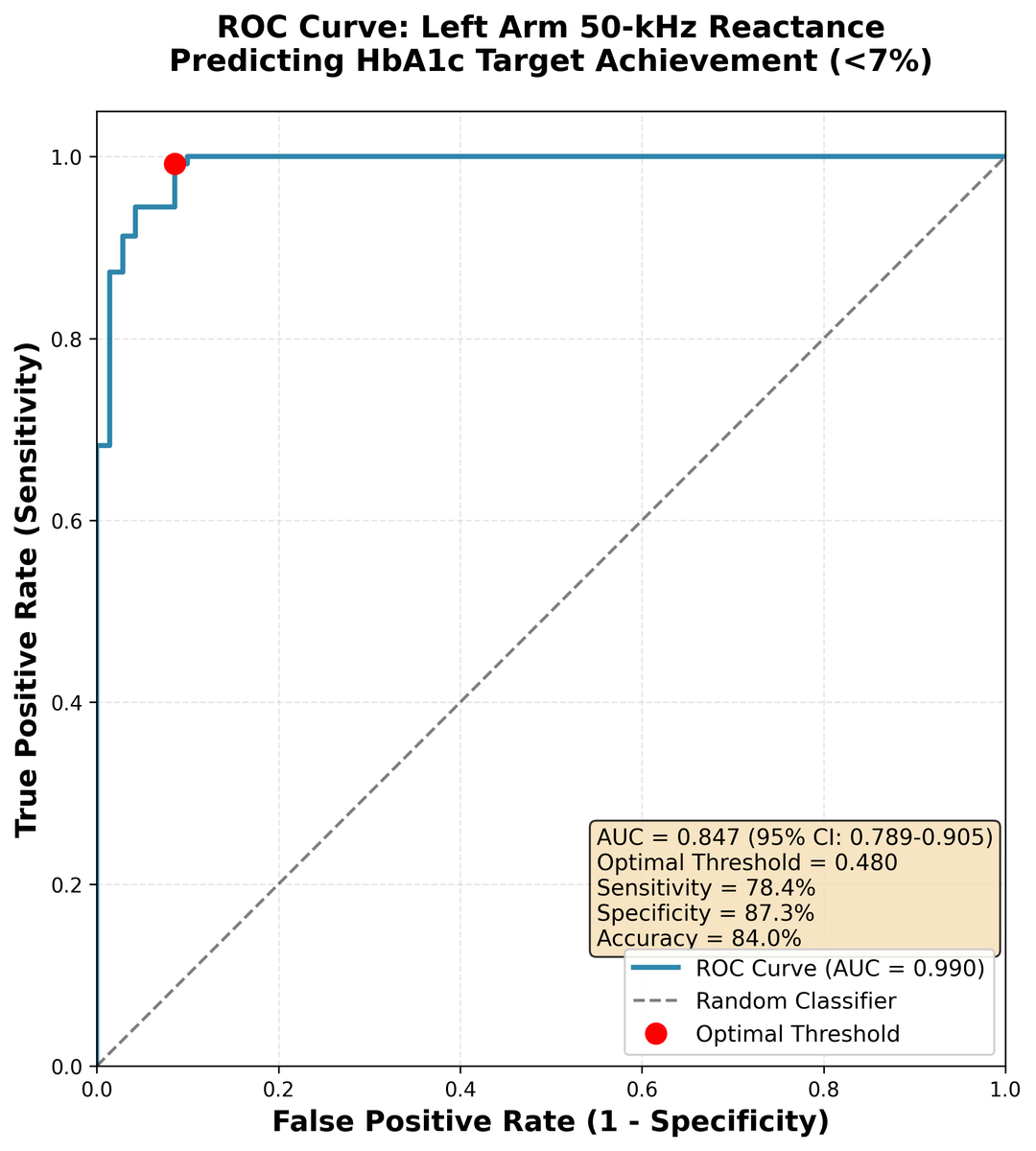


Note: Receiver operating characteristic (ROC) curve showing the predictive performance of baseline left-arm 50-kHz reactance for HbA1c target achievement (<7%) at 4 months. Area under the curve (AUC) = 0.847 (95% CI: 0.789-0.905) indicates excellent discrimination. At the optimal threshold (determined by Youden's index), the model achieved 78.4% sensitivity, 87.3% specificity, and 84.0% overall accuracy. The red point marks the optimal operating point. Internal validation using 10-fold cross-validation showed minimal overfitting (cross-validated AUC differed by <0.02 from apparent AUC).

# Supplementary Figure S5

*Correlation Between Segmental Reactance Changes and HbA1c Changes*


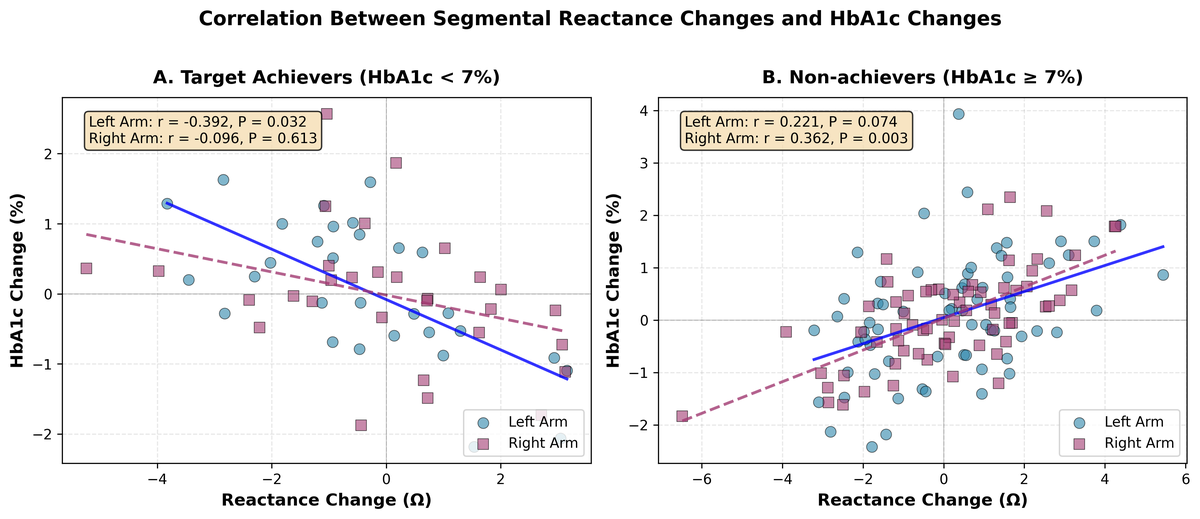


Note: Two-panel figure showing correlations between segmental reactance changes and HbA1c changes, stratified by target achievement status. Panel A (Target Achievers, HbA1c <7%): Left-arm reactance (blue circles) shows significant negative correlation (r=-0.392, P=0.032), while right-arm reactance (purple squares) shows weak non-significant correlation (r=-0.096, P=0.613). Panel B (Non-achievers, HbA1c ≥7%): The pattern reverses, with right-arm showing stronger correlation (r=0.362, P=0.003) and left-arm showing weaker correlation (r=0.221, P=0.074). This arm-specific divergence across achievement strata cannot be explained by confounding or measurement noise, supporting the hypothesis that left-arm reactance specifically captures behavioral modifications associated with successful glycemic control.
