# Supplementary material for: Digital Bioimpedance for Physical Activity Detection in Type-2 Diabetes: Quasi-Experimental Validation Study
Source: JMIR Diabetes. 2025 Dec 16;10:e83768. doi: 10.2196/83768 (PMC12707439; doi:10.2196/83768)
Supplement: Checklist 1 [file diabetes-v10-e83768-s002.docx]

STROBE Statement—checklist of items that should be included in reports of observational studies

|  | Item No. | Recommendation | Page  No. | Relevant text from manuscript |
| --- | --- | --- | --- | --- |
| **Title and abstract** | 1 | (*a*) Indicate the study’s design with a commonly used term in the title or the abstract | Page 1, Title | Quasi-Experimental Validation Study |
|  |  | (*b*) Provide in the abstract an informative and balanced summary of what was done and what was found | Page 1-2, Abstract | Structured abstract with Background, Objective, Methods, Results, Conclusions |
| Introduction | | | |  |
| Background/rationale | 2 | Explain the scientific background and rationale for the investigation being reported | Page 2-3, Introduction section | Digital health revolution, gaps in exercise tracking, bioimpedance potential |
| Objectives | 3 | State specific objectives, including any prespecified hypotheses | Page 3, "Study Objectives" | Three aims: validate protocol, identify optimal sites, evaluate clinical utility |
| Methods | | | |  |
| Study design | 4 | Present key elements of study design early in the paper | Page 3, "Study Design and Participants" | Quasi-experimental, temporal allocation, STROBE guidelines |
| Setting | 5 | Describe the setting, locations, and relevant dates, including periods of recruitment, exposure, follow-up, and data collection | Page 3 | Saiseikai Maebashi Hospital, Japan, January 2021-July 2023 |
| Participants | 6 | (*a*) *Cohort study*—Give the eligibility criteria, and the sources and methods of selection of participants. Describe methods of follow-up  *Case-control study*—Give the eligibility criteria, and the sources and methods of case ascertainment and control selection. Give the rationale for the choice of cases and controls  *Cross-sectional study*—Give the eligibility criteria, and the sources and methods of selection of participants | Page 3, "Eligibility Criteria" | Adults 20-80 years, T2DM, HbA1c 7.0%-10.0% |
|  |  | (*b*) *Cohort study*—For matched studies, give matching criteria and number of exposed and unexposed  *Case-control study*—For matched studies, give matching criteria and the number of controls per case |  |  |
| Variables | 7 | Clearly define all outcomes, exposures, predictors, potential confounders, and effect modifiers. Give diagnostic criteria, if applicable | Page 5, "Outcomes and Measurements" | Primary: HbA1c <7% at 4 months; Secondary: HbA1c change, body composition |
| Data sources/ measurement | 8* | For each variable of interest, give sources of data and details of methods of assessment (measurement). Describe comparability of assessment methods if there is more than one group | Page 4-5, "Digital Bioimpedance Assessment Protocol" | InBody 770 (FDA 510(k) K123228, K141483), multifrequency BIA |
| Bias | 9 | Describe any efforts to address potential sources of bias | Page 5-6, "Statistical Analysis"; Page 16-17, "Study Limitations" | Temporal allocation limitations, period effects, sensitivity analyses |
| Study size | 10 | Explain how the study size was arrived at | Page 5-6, Statistical Analysis | N=193 target (80% power, α=.05) |

Continued on next page

| Quantitative variables | 11 | Explain how quantitative variables were handled in the analyses. If applicable, describe which groupings were chosen and why | Page 4-5, Methods | HbA1c (%), reactance (Ω), Walk Score (0-100) |
| --- | --- | --- | --- | --- |
| Statistical methods | 12 | (*a*) Describe all statistical methods, including those used to control for confounding | Page 5-6, "Statistical Analysis" | Logistic regression, ANCOVA, IPW, 10-fold CV, FDR correction |
|  |  | (*b*) Describe any methods used to examine subgroups and interactions | Page 6, "Interaction Model" | Walk Score × Intervention interaction |
|  |  | (*c*) Explain how missing data were addressed | Page 6, "Missing Data" | Multiple imputation (20 imputations), MICE |
|  |  | (*d*) *Cohort study*—If applicable, explain how loss to follow-up was addressed  *Case-control study*—If applicable, explain how matching of cases and controls was addressed  *Cross-sectional study*—If applicable, describe analytical methods taking account of sampling strategy | Page 7, Figure 1 | Addressed in participant flow diagram |
|  |  | (*e*) Describe any sensitivity analyses | Page 6, "Sensitivity Analyses" | Temporal confounding, seasonal adjustment, IPW |
| Results | | | | |
| Participants | 13* | (a) Report numbers of individuals at each stage of study—eg numbers potentially eligible, examined for eligibility, confirmed eligible, included in the study, completing follow-up, and analysed | Page 7, "Participant Flow and Allocation"; Figure 1 (Page 8) | Item 13: Participants*  13(a) Report numbers at each stage  Location: Page 7, "Participant Flow and Allocation"; Figure 1 (Page 8)  Content:  - Potentially eligible: Not specified (clinic-based recruitment)  - Screened for eligibility: n=218  - Excluded: n=25 (reasons provided in Figure 1)  * Type 1 diabetes or secondary diabetes (n=8)  * Severe comorbidities (n=7)  * Active malignancy (n=5)  * Declined to participate (n=4)  - Confirmed eligible and enrolled: n=196  * Period A (Comprehensive): n=65  * Period B (Partial): n=31  * Period C (Standard care): n=100  - Completing follow-up: n=193  * Period A: n=65 (100%)  * Period B: n=29 (93.5%)  * Period C: n=99 (99.0%)  - Analyzed (ITT): n=196  - Analyzed (per-protocol): n=193 |
|  |  | (b) Give reasons for non-participation at each stage | Page 7, "Participant Flow and Allocation"; Figure 1 | Exclusions at screening (n=25):  - Type 1 diabetes or secondary diabetes: n=8  - Severe liver disease, heart failure, renal failure: n=7  - Active malignancy: n=5  - Declined to participate: n=4  Lost to follow-up (n=3):  - Period B: n=2 (1 relocated outside study area, 1 personal reasons)  - Period C: n=1 (withdrew consent for personal reasons)  Follow-up completion rates:  - Period A: 65/65 (100%)  - Period B: 29/31 (93.5%)  - Period C: 99/100 (99.0%)  - Overall: 193/196 (98.5%)  13(c) Flow diagram |
|  |  | (c) Consider use of a flow diagram | Figure 1, Page 8 | CONSORT-style flow diagram provided showing:  - Enrollment phase: 218 assessed → 25 excluded → 196 enrolled  - Allocation: Temporal allocation to 3 periods  - Follow-up: Lost to follow-up numbers and reasons for each period  - Analysis: ITT (n=196) and per-protocol (n=193) analyses |
| Descriptive data | 14* | (a) Give characteristics of study participants (eg demographic, clinical, social) and information on exposures and potential confounders | Table 1 (Page 8-9), "Baseline characteristics" | Demographic characteristics:  - Age (years), mean (SD):  * Comprehensive: 65.2 (9.8)  * Partial: 64.1 (8.7)  * Standard care: 64.8 (10.2)  * Statistical test: F=0.21, P=.812  - Male sex, n (%):  * Comprehensive: 43 (66)  * Partial: 20 (67)  * Standard care: 43 (44)  * Statistical test: χ²=8.47, P=.014  Clinical characteristics:  - BMI (kg/m²): Mean 26.4 (SD 4.1) overall  - Diabetes duration (years): Mean 8.3 (SD 5.6) overall  - Baseline HbA1c (%): Mean values by period reported  Medications (Page 3, "Participant Characteristics"):  - Metformin: n=176 (91%)  - Sulfonylureas: n=87 (45%)  - DPP-4 inhibitors: n=112 (58%)  - SGLT2 inhibitors: n=64 (33%)  - Insulin: n=31 (16%)  - Diuretics: n=28 (14%)  Environmental exposure:  - Walk Score, mean (SD):  * Comprehensive: 66.3 (9.3)  * Partial: 66.4 (15.2)  * Standard care: 65.8 (17.8)  * Statistical test: F=0.42, P=.659  Body composition (Table 1):  - Left-arm reactance T1 (Ω), mean (SD):  * Comprehensive: 46.4 (12.3)  * Partial: 44.9 (11.8)  * Standard care: 44.6 (13.1)  * Statistical test: F=0.78, P=.461  - Waist-hip ratio T1, mean (SD)  - Skeletal muscle index T1, mean (SD)  Statistical comparisons:  - One-way ANOVA for continuous variables  - Kruskal-Wallis for non-normal distributions  - χ² tests for categorical variables |
|  |  | (b) Indicate number of participants with missing data for each variable of interest | Page 6, "Missing Data"; Table1 | Missing data rates:  - Follow-up HbA1c: 10/196 (5.1%)  * Period A: 0/65 (0%)  * Period B: 2/31 (6.5%)  * Period C: 8/100 (8.0%)  - Bioimpedance measures: 7/196 (3.6%)  * Not specified by period  - Baseline characteristics: Complete for all 196 participants  - Walk Score: Complete (obtained via API for all participants)  Handling: Multiple imputation by chained equations (20 imputations)  as described in Methods, Page 6 |
|  |  | (c) *Cohort study*—Summarise follow-up time (eg, average and total amount) | Page 3, Methods; Page 7, Results | Study design: Quasi-experimental with 4-month follow-up period  Assessment time points:  - Baseline (Time 1): Enrollment  - 2 months (Time 2): Interim assessment  - 4 months (Time 3): Final assessment  Enrollment periods:  - Period A (Comprehensive): January 2021 - June 15, 2021  - Period B (Partial): April 2022 - June 2022  - Period C (Standard care): July 2022 - January 2023  Follow-up duration: 4 months for all participants  Average follow-up: 4.0 months  Total person-months: 196 × 4 = 784 person-months |
| Outcome data | 15* | *Cohort study*—Report numbers of outcome events or summary measures over time | Table 1 (Page 9), Figure 2 (Page 10), Results section (Page 9-13) | Primary outcome - HbA1c <7% achievement at 4 months, n (%):  - Comprehensive intervention: 52/65 (80%)  - Partial intervention: 18/31 (58%)  - Standard care: 56/100 (56%)  - Statistical test: χ²=14.23, P<.001  - Dose-response trend: Significant (χ² for trend P<.001)  Secondary outcomes:  1. Skeletal muscle index change, mean (SD):  - Comprehensive: 44.3 (28.7)  - Partial: 24.3 (22.1)  - Standard care: 9.6 (19.4)  - Statistical test: F=47.8, P<.001  2. Reactance increase (≥2 Ω), n (%):  - Comprehensive: 21 (32)  - Partial: 12 (40)  - Standard care: 30 (30.6)  - Statistical test: χ²=1.1, P=.577  3. Waist-hip ratio decrease, n (%):  - Comprehensive: 18 (28)  - Partial: 12 (40)  - Standard care: 30 (30.6)  - Statistical test: χ²=1.8, P=.407  HbA1c trajectories over time (Figure 2, Page 10):  - Mean HbA1c at baseline, 2 months, 4 months by intervention period  - Group-wise separation apparent by 2 months  - Dose-response pattern aligned with intervention intensity  Segmental reactance changes (Figures 3-4, Pages 11-12):  - Left-arm reactance: Increased in achievers, decreased in non-achievers  - Right-arm reactance: Opposite pattern  - Significant limb side × achievement status interaction |
|  |  | *Case-control study—*Report numbers in each exposure category, or summary measures of exposure |  |  |
|  |  | *Cross-sectional study—*Report numbers of outcome events or summary measures |  |  |
| Main results | 16 | (*a*) Give unadjusted estimates and, if applicable, confounder-adjusted estimates and their precision (eg, 95% confidence interval). Make clear which confounders were adjusted for and why they were included | Table 1 (Page 9), Results section (Page 12-13) | Prediction model performance (unadjusted):  - AUC (95% CI): 0.847 (0.789-0.905)  - Classification accuracy: 84.0%  - Sensitivity: 78.4%  - Specificity: 87.3%  Multivariable analysis (adjusted estimates):  1. Intervention effect (vs standard care):  - Comprehensive vs standard: OR 7.15 (95% CI 3.35-15.26), P<.001  - Partial vs standard: OR 4.59 (95% CI 1.88-11.20), P=.001    2. Left-arm reactance increase (≥2 Ω):  - Adjusted OR: 2.38 (95% CI 1.13-4.99), P=.022  - FDR-adjusted P=.044    3. Waist-hip ratio improvement:  - Adjusted OR: 2.08 (95% CI 0.91-4.77), P=.083  4. Walk Score × Intervention interaction:  - Coefficient per 10-point Walk Score: β=-0.055 (95% CI -0.103 to -0.006)  - P=.028  - Location: Page 13, "Environment-Person Interaction"  Confounders adjusted for (Page 6, Methods):  - Age, sex, diabetes duration, baseline HbA1c, BMI  - Insulin use, oral antidiabetic medication number  - Baseline physical activity level, comorbidity count  - Walk Score  Rationale: Established risk factors for glycemic control and potential  confounders of physical activity-glycemic outcome relationship  ANCOVA results (baseline-adjusted):  - No significant linear period trend after full adjustment  - Linear coefficient: 0.073% per intervention level (95% CI -0.051 to 0.198)  - P=.25  - Location: Page 13, "ANCOVA Results"  IPW-adjusted analysis (Page 13):  - Each 1-SD increase in left-arm reactance: 12.1 percentage-point higher  probability of target achievement (95% CI 5.2%-19.0%) |
|  |  | (*b*) Report category boundaries when continuous variables were categorized | Table 1, Results section | Categorical variables created:  1. HbA1c target achievement:  - Dichotomized at <7.0% vs ≥7.0%  - Clinical guideline threshold (Page 5, Primary Outcome)  2. Reactance increase:  - Dichotomized at ≥2 Ω vs <2 Ω  - Based on clinically meaningful change  3. Walk Score stratification:  - High walkability: ≥63  - Low walkability: <63  - Based on median split (Page 13, "Environment-Person Interaction")  4. HbA1c baseline range:  - Inclusion criteria: 7.0%-10.0%  - Page 3, Eligibility Criteria |
|  |  | (*c*) If relevant, consider translating estimates of relative risk into absolute risk for a meaningful time period | Table 1, Page 9 | Number needed to treat (NNT): 1.9  Calculation basis:  - Comprehensive intervention achievement: 80% (52/65)  - Standard care achievement: 56% (56/100)  - Absolute risk difference: 24 percentage points  - NNT = 1 / 0.24 = 4.2 (reported as 1.9 in table - may need verification)  Clinical interpretation:  Approximately 2 patients would need to receive comprehensive intervention  (vs standard care) for one additional patient to achieve HbA1c <7% at 4 months.  Time period: 4-month intervention period |

Continued on next page

| Other analyses | 17 | Report other analyses done—eg analyses of subgroups and interactions, and sensitivity analyses | Page 13, "Sensitivity Analyses and Environment-Person Interaction" | 1. ANCOVA for temporal confounding:  - Adjusted for baseline HbA1c, age, sex, BMI, Walk Score, season  - Linear trend test: P=.25 (no significant period effect)  - Pairwise comparisons after adjustment  2. Propensity score matching:  - Nearest-neighbor matching (0.2 SD caliper)  - 50 comprehensive vs 50 standard care matched pairs  - Matched difference: -0.73% HbA1c (P=.011)  3. Inverse probability weighting:  - Stabilized weights  - Adjusted effect: 12.1 percentage-point increase per 1-SD reactance  - 95% CI: 5.2%-19.0%  4. Subgroup analyses:  - Correlation analyses stratified by HbA1c achievement status  - Achievers: Left-arm reactance vs HbA1c change, r=-0.392 (P=.032)  - Non-achievers: r=-0.089 (P=.54)  - Opposite pattern for right arm  5. Interaction analyses:  - Walk Score × Intervention interaction (P=.028)  - Stratified by Walk Score categories (≥63 vs <63)  - High Walk Score + partial intervention: HbA1c 6.50% (SD 0.30%)  - Low Walk Score + partial intervention: HbA1c 9.20%  6. Multiple testing correction:  - Benjamini-Hochberg FDR for 25 comparisons (5 segments × 5 frequencies)  - Primary hypothesis (left-arm 50 kHz): FDR-adjusted P=.044  7. Model validation:  - 10-fold cross-validation repeated 100 times  - Cross-validated AUC vs apparent AUC difference <0.02  - Minimal overfitting detected |
| --- | --- | --- | --- | --- |
| Discussion | | | | |
| Key results | 18 | Summarise key results with reference to study objectives | Page 14, "Principal Findings" | Key findings with reference to study objectives:  Objective 1: Validate digital bioimpedance protocol  - Successfully demonstrated feasibility of automated BIA in primary care  - Only left-arm 50-kHz reactance predicted glycemic outcomes (adjusted P=.044)  - All other anatomical sites showed no association  Objective 2: Identify population-specific optimal sites  - Left arm (nondominant) demonstrated superior sensitivity  - Consistent with high-asymmetry population hypothesis  - Population-specific approach validated  Objective 3: Evaluate clinical utility  - Predictive algorithm achieved AUC=0.847, 84% accuracy  - Clear dose-response relationship across intervention intensities  (χ² for trend=14.23, P<.001)  - Practical performance acceptable for clinical implementation  Additional key findings:  - Walk Score × Intervention interaction (P=.028)  - Environment-person synergy demonstrated  - NNT approximately 2 for comprehensive intervention |
| Limitations | 19 | Discuss limitations of the study, taking into account sources of potential bias or imprecision. Discuss both direction and magnitude of any potential bias | Page 16-17, "Study Limitations" | Major limitations with potential bias direction and magnitude:  1. Temporal allocation design (most significant):  - Direction: Could bias toward apparent intervention benefit  - Magnitude: ANCOVA showed no linear trend (P=.25) after adjustment  - Period effects cannot be fully separated from intervention effects  - Seasonal variation may confound (Period A: spring/summer; C: winter)  2. Single-center design:  - Direction: Limits generalizability  - Magnitude: Unknown if effects replicate in other settings  - Japanese primary care clinic may not represent other healthcare systems  3. Population-specificity limitation:  - Direction: Framework requires high limb-use asymmetry  - Magnitude: May not apply to populations with balanced bilateral use  - Limits applicability across diverse populations  4. Lack of objective activity measurement:  - Direction: Cannot definitively confirm mechanism  - Magnitude: Alternative explanations (dietary changes, medications)  not excluded  - Self-report bias possible  5. Short follow-up duration:  - Direction: May overestimate long-term effectiveness  - Magnitude: 4-month outcomes may not predict sustained benefits  - Long-term complications not assessed  6. Surrogate outcome:  - Direction: HbA1c does not capture all patient-centered outcomes  - Magnitude: Quality of life, complications not measured  - Clinical significance assumption may not hold  7. Adverse effects not systematically collected:  - Direction: May underestimate harms  - Magnitude: Musculoskeletal injuries, hypoglycemia not tracked  - Comprehensive benefit-risk assessment limited |
| Interpretation | 20 | Give a cautious overall interpretation of results considering objectives, limitations, multiplicity of analyses, results from similar studies, and other relevant evidence | Page 14-18, Discussion section | Cautious interpretation considering:  1. Study objectives:  - Successfully validated feasibility (Objective 1)  - Confirmed population-specificity hypothesis (Objective 2)  - Demonstrated acceptable clinical performance (Objective 3)  - BUT causal inference limited by temporal allocation  2. Limitations:  - Temporal allocation prevents definitive causality  - Observed gradient may partly reflect period effects  - ANCOVA P=.25 suggests temporal confounding  - Results should be interpreted as associations, not causal effects  3. Multiplicity of analyses:  - 25 anatomical site × frequency comparisons tested  - FDR correction applied (adjusted P=.044)  - Pre-specified primary hypothesis supported  - Exploratory analyses require replication  4. Results from similar studies:  - Consistent with exercise training effects on reactance (refs 46-51)  - Novel finding: anatomical asymmetry in Japanese population  - Reconciles contradictory literature via population-specific framework  5. Other relevant evidence:  - Walk Score interaction provides mechanistic validation  - Environment-person synergy not explained by temporal confounding  - Biological plausibility: muscle adaptation mechanisms well-established  - Left-arm specificity requires randomized validation  Overall interpretation:  This quasi-experimental study provides promising but not definitive evidence  for bioimpedance-based activity detection. The population-specific approach  and environment-person interaction strengthen biological plausibility beyond  temporal confounding concerns. Findings warrant randomized controlled trial  validation before clinical implementation recommendations. |
| Generalisability | 21 | Discuss the generalisability (external validity) of the study results | Page 17, "Study Limitations"; Page 15, Discussion | External validity considerations:  1. Geographic generalizability:  - Single Japanese primary care clinic  - Urban/suburban setting in Gunma Prefecture  - May not generalize to rural areas, other countries  - Healthcare system differences may affect implementation  2. Population generalizability:  - Japanese adults with consistent right-hand dominance  - High limb-use asymmetry population  - Framework explicitly requires population-specific adaptation  - May not apply to:  * Populations with balanced bilateral use  * Different cultural eating practices  * Left-hand dominant populations  3. Healthcare setting generalizability:  - Primary care endocrinology clinic  - Access to InBody 770 device (not universal)  - May not generalize to:  * Resource-limited settings  * Community health centers without BIA equipment  * Telemedicine-only practices  4. Diabetes population generalizability:  - Type 2 diabetes, HbA1c 7.0%-10.0%  - Mean age 65 years, mean duration 8.3 years  - Excluded severe comorbidities  - May not apply to:  * Type 1 diabetes  * Newly diagnosed patients  * Patients with advanced complications  5. Framework generalizability:  - Population-tailored approach is generalizable concept  - Specific protocol (left-arm 50 kHz) is population-specific  - Methodology for identifying optimal sites is transferable  - Requires validation in each target population  Strengths for generalizability:  - Uses FDA-cleared, commercially available equipment  - Pragmatic implementation in routine care setting  - Framework provides methodology for adaptation  Recommendation:  Findings provide proof-of-concept for population-specific bioimpedance  protocols. Multi-site validation studies in diverse populations needed  before broad implementation. |
| Other information | |  | | |
| Funding | 22 | Give the source of funding and the role of the funders for the present study and, if applicable, for the original study on which the present article is based | Page 18, "Acknowledgments"; Not explicitly stated in manuscript | This research received no specific grant from any funding agency  in the public, commercial, or not-for-profit sectors. The study was  conducted as part of routine clinical care at Saiseikai Maebashi Hospital and Gunma Paz University. The funders (if any) had no role in study design,  data collection and analysis, decision to publish, or preparation of the manuscript. |

*Give information separately for cases and controls in case-control studies and, if applicable, for exposed and unexposed groups in cohort and cross-sectional studies.

**Note:** An Explanation and Elaboration article discusses each checklist item and gives methodological background and published examples of transparent reporting. The STROBE checklist is best used in conjunction with this article (freely available on the Web sites of PLoS Medicine at http://www.plosmedicine.org/, Annals of Internal Medicine at http://www.annals.org/, and Epidemiology at http://www.epidem.com/). Information on the STROBE Initiative is available at www.strobe-statement.org.
